# Supplementary material for: Carbon-Nanotube-Based Nanocomposites in Environmental Remediation: An Overview of Typologies and Applications and an Analysis of Their Paradoxical Double-Sided Effects
Source: J Xenobiot. 2025 May 21;15(3):76. doi: 10.3390/jox15030076 (PMC12101166; doi:10.3390/jox15030076)
Supplement: Supplementary file 1 [file jox-15-00076-s001.zip › jox-3606725-supplementary.pdf]

Supplementary Materials

Carbon Nanotube-Based Nanocomposites in Environmental Remediation: An Overview of Typologies, Applications and an Analysis of Their Paradoxical Double-Sided Effects

Silvana Alfei [1,*], and Guendalina Zuccari [1,2,*]

[1] Department of Pharmacy (DIFAR), University of Genoa, Viale Cembrano, 4, 16148 Genoa, Italy
[2] Laboratory of Experimental Therapies in Oncology, IRCCS Istituto Giannina Gaslini, Via G. Gaslini 5, 16147 Genoa, Italy
* Correspondence: alfei@difar.unige.it; Tel.: +39 010 355 2296 (S.A.); guendalina.zuccari@unige.it (G.Z.)

Tables

The list of references reported in Tables is available under the last Table S11.

Table S1. Main methods to synthesize carbon nanotubes (CNTs).

Method	Invention paternity/description	Type of tube	Advantages/Disadvantages	Refs
AD	(1991) From procedure to get fullerenes applying a current (110 A) at T >1,700°C<br>CNTs form in the carbon soot of GR electrode	N.R.	⬇Structural defects<br>Macroscopic production	[1–3]
LA	Dr. Richard Smalley*<br>Graphite is blasted with a laser<br>Graphite and cobalt/nickel (metal catalyst particles) blasted with a laser	MWCNTs<br>SWCNTs	N.R.	[4,5]<br>[6]<br>[7]
CVD	Substrate of nickel, cobalt, iron, or combination catalyst NPs<br>Heating at 700°C under a flux of a "process gas" ** and a "carbon-containing gas" *** to promote CNTs growing<br>CNTs directly growth on the desired substrate by careful deposition of the catalyst	N.R.	⬇ Cost, scaling up<br>Industrial production<br>If present, need to remove the catalyst support via acid treatment, with possible damage to the CNT structure	[8–13]<br>[8,14]
PECVD	Advanced CVD consisting of plasma generation by applying a strong electric field during CNTs growth	VACNTs	N.R.	[15]
HiPco	Rice University<br>HPCO reacts with FePC, Fe NPs form providing the nucleation surface where CNTs form by the transformation of CO into carbon	SWCNTs	Production from milligrams to grams scale No environmental release of wastes	[4,5]
SGCVD	Kenji Hata, Sumio Iijima at AIST (Japan)<br>Introduction of water into the CVD reactor	VANTAs<br>Forest material	⬆Activity and lifetime of the catalyst<br>⬆To LA and HiPco by 100 times<br>⬇ Mass density, >99.98% pure, 2.5 mm height SWNT 10min<br>Easy separation of CNTs from the catalyst<br>>1 mm long VANTAs in several shapes §	[16]<br>[5,17]
PT	Olivier Smiljanic (2000), Institut National de la Recherche Scientifique (INRS), Varennes (Canada)<br>Argon, ethylene and ferrocene into PLASMOTRON, thus developing an intense 'flame' containing CNTs, metallic, carbon NPs and amorphous carbon	SWCNTs	10-fold ⬇consumed energy than in LA or AD.	[5,18]
ITP	Sherbrooke University and the National Research Council of Canada<br>Modified PT procedure<br>TP is generated by HFOCs in a loop and is conserved in flowing inert gas	SWCNTs	CNTs with different diameter distributions	[19]

LEM	Metal ions reduced to metal forms on cathode provide the nucleation surface for CNTs growth which derive from electrolysis of molten carbonates #	MWCNTs	↑Valued CNTs<br>Strategy for CO_2 capture and conversions	[5,20,21]
NICFE	CNTs form naturally in flames emitted by burning methane, ethylene, benzene		↑Irregular in dimensions and quality CNT	[22–24]
ACVD	CNTs are synthesized in the gas phase and deposited in the form of randomly oriented networks, ready for many applications including transparent conductors.	SWCNTs<br>TF	Clean defect less SWCNTs with limited yield	[25–27]

PR = Parameters; Adv = advantages; Disadv = disadvantages; * Rice University; AD = arc discharge; LA = laser ablation; CVD 0 chemical vapor deposition; ↓ = less, minor, low; ** ammonia, nitrogen, or hydrogen; *** acetylene, ethylene, ethanol, or methane; PECVD = plasma-enhanced chemical vapor deposition; VACNTs = vertically aligned CNTs; HiPco = High-Pressure Carbon Monoxide Process; HPCO = high-pressure carbon monoxide; FePC = iron pentacarbonyl; SGCVD = super-growth CVD, also known as water-assisted chemical vapor deposition; VANTAs = millimetre-high vertically aligned nanotube arrays; "forests" materials = tubes aligned to the substrate; ACVD = aerosol CVD; TF = thin films; ↑ = enhanced, improved, high, higher, highly, superior; § = sheets and bars, by applying weak compression during the process; PT = plasma torch; PLASMOTRON = microwave plasma torch; LEM = liquid electrolysis method; NICFE = natural, incidental, and controlled flame environments; GR = graphene; HFOCs = high-frequency oscillating currents; TP = thermal plasma; # the reactant is a carbon dioxide greenhouse gas.

Table S2. Most relevant research articles on CNT synthesis from biomass by different methods, operating parameters and main properties.

Feed stock	Method	Support/Catalyst	Operating parameters			CNTs	Properties				Refs.
			T (°C)	Time (min)	Carrier gas		SA (m^2/g)	D (nm)	*(%)	L (μm)	
Rice straw	P	Al_2O_3/Fe-Ni	830	30	N_2	MWCNT	188	15–40	N.A./N.A.	N.A.	[28]
Olive oil	P	Si wafer/$NiCl_2$	900	60	Ar	SWCNT	N.A.	27–31	N.A./ N.A.	N.A.	[29]
Turpentine oil	P	Zeolite/Fe-Co	850	25	N_2	SWCNT	N.A.	7–20	58/N.A.	N.A.	[30]
Coconut oil	CVD	Fe	850	60	N_2	MWCNT	N.A.	80–100	58/N.A.	3–4	[31]
Sesame oil	P+CVD	FNS/CH_3CN	900	15	Ar	MWCNT	N.A.	30–60	58/N.A.	3–4	[32]
Palm oil	CVD	Silicon/Ferrocene	750	30	Ar	SWCNT<br>MWCNT	N.A.	0.6–1.2	90/N.A.	110	[33]

Plastic waste	P+CVD	Ni	800	30	N_2	MWCNT	N.A.	40–50	N.A./31	N.A.	[34]
Petroleum coke	CVD	Silica/Fe	700	60	He	MWCNT	N.A.	18	N.A./N.A.	N.A.	[35]
Plastic waste	P	Cordierite/*N*-Mg	750	60	N_2	MWCNT	N.A.	30–50	N.A./93	30–50	[36]
NCG	P+CVD	Ni/Al_2O_3	1000	112	N_2	SWCNT	N.A.	10	N.A./82	N.A.	[37]
Ethylene	FC, CVD	Co/Fe, Co/Ni, Ni, Co, Fe	1050	0.17	N_2	SWCNT	N.A.	0.67–2	61–69/ N.A.	N.A.	[38]
Rice straw	T-CVD	Fe, Ni	800	120	N_2	MWCNT	20 (CNT+Fe)35 (CNT+Fe+Ni)	22 (CNT+Fe)66 (CNT+Fe+Ni)	N.A./41–44	N.A.	[39]

*Purity/yield; CH_3CN = acetonitrile; N.A. = information not available; D = Diameter; P = pyrolysis; SA = surface area; T = temperature; NCG = natural condensed gas; floating catalyst; CVD = chemical vapor deposition; T-CVD = thermal CVD; FNS = ferrocene nitrogen source.

Table S3. Preparation of CNTs from biomass via microwave irradiation.

Raw material	Microwave conditions	Product	Properties of CNTs	Refs
Sugarcane bagasse	600 W, 500 °C	NPs and CNTs	Average Ø = 20-50 nm	[40]
Waste rice husk	900 W, 2.45 GHz	GR CNTs + GR-CNTs	L = tens of μm, Ø = 50-200 nm	[41]
Wheat straw, oat husk, rapeseed cake and hazelnut hulls	200 W, 2.45 GHz, 80 °C, 17 psi	CNTs	Ø = 17-100 nm	[42]
Palm kernel shells	2000 W, 2.45 GHz, 600 °C	MWCNTs	Ø = 50-100 nm	[43]
Pine nut shells	2000 W, 600 °C	MWCNTs	L = 2600-3200 nm	[44,45]
Gumwood	300 W, 500 °C, 2.45 GHz	MWCNTs	WT = 5-7 nm, Ø about 50 nm	

L = length; Ø = diameter; NPs = nanoparticles; Gr = graphene; WT = wall thickness.

Table S4. Reported applications of CNTs and modified CNTs for absorption of several heavy metals from water by different mechanisms.

Target pollutant	Adsorbents	Adsorption Mechanism	pH, CT, AD*	Refs.
**Hg (II)**	ATPPBr-CNT	IE/complexation	pH 5.5, 28 min, 5.5 mg	[46]

	TBABr-Gly-CNT	Physisorption	pH 6.4, 45 min, 6.0 mg	[47]
	MWCNTs-AA	Chemisorption	pH 6.0, 180 min, 10 mg	[48]
	CNTs- MnO_2	EI	pH 5–7, 80 min, 20 mg	[49]
	SWCNT-Thiol (SH)	Chemisorption	pH 5.0, 60 min, 250 mg	[50]
	MWCNTs-Sulphur	SASBI, EI	pH 12.15, 60 min, 100 mg	[51]
	O-MWCNTs	Chemisorption, ED, IE, physisorption, complexation	pH 6.0, 90 min, 20 mg	[52]
	CNTs-Iodide	Chemisorption, ED, IE, physisorption, complexation, SASBI	pH 6.0, 90 min, 20 mg	[52]
	MWCNTs-Sulphur	IE, physisorption, chemisorption, ED, complexation, SASBI	pH 6.0, 90 min, 20 mg	[52]
	Amino, SH-MWCNTs	Physisorption, SASBI	pH 6.0, 60 min, 400 mg	[53]
	Raw MWCNTs	EI, complexation, chemisorption, IPD	pH 3.5, 10 mg	[54]
	MWCNTs-C_6H_5OH	ED	pH 1.2, 10 mg	[54]
	MWCNTs-COOH	ED	pH < 1.0, 10 mg	[54]
	MWCNTs	Chemisorption	pH 7.0, 500 mg, 120 min	[55]
	PAAA/GO-SWCNTs	Chemisorption	pH 5.3, 210 mg, 6.87 min	[56]
	MWCNTs-COOH	Chemisorption, EI	pH 7.0, 500 mg, 90 min	[57]
	MWCNTs-OH	Chemisorption, EI	pH 7.0, 500 mg, 90 min	[57]
	MWCNTs-NH_2	Chemisorption, EI	pH 7.0, 500 mg, 90 min	[57]
	MWCNTs-3-AP	Chemisorption, physisorption	pH 7.7, 20 mg, 120 min	[58]
	CNTs-SH/Fe_3O_4	Chemisorption, physisorption	pH 6.0, 500 mg, 90 min	[59]
	CuS-MWCNTs	Chemisorption, physisorption	50 mg,120 min	[60]
	MWCNTs-KOH@NiNPs	Chemisorption	pH 5.5, 40 mg, 30 min	[61]
	CNTs-DES	Chemisorption, EI	pH 5, 5 mg, 15 min	[62]
**Pb (II)**	T2AEA-MWCNTs	Chemisorption, physisorption	pH 6.6, 10 mg, 45 min	[63]

	M-MWCNTs- 8-aminoquinline	Chemisorption	pH 6.4, 25 mg, 5 min	[64]
	SWCNTs-WSh	Chemisorption, precipitation	pH 5, 1000 mg, 30 min	[65]
	As-produced MWCNT	Chemisorption, EI	pH 5.5, 20 mg 120 min	[66]
	O-MWCNTs	Chemisorption, EI	pH 5.5, 1000 mg 120 min	[66]
	O-MWCNTs	EI	pH 5, 5 mg, 30 min	[67]
	MWCNTs-isocyanate	EI	pH 6, 5 mg, 30 min	[68]
	PP-MWCNTs	Chemisorption, physisorption	pH 6, 60 mg, 60 min	[69]
	N_2H_4-SH-Fe_3O_4/O-MWCNTs	Chemisorption, physisorption	pH 6, 40 mg, 30 min	[70]
	Fe_3O_4/O-MWCNTs	Chemisorption, complexation, EI	pH 6, 1000 mg, 360 min	[71]
	PAMAM-CNTs	Physisorption, EI	pH 7, 30 mg, -	[72]
	Acidified MWCNTs	Chemisorption, complexation, IE	pH 9, 500 mg, 600 min	[73]
	Fe_3O_4-CNT	Chemisorption, physisorption	pH 6, 100 mg, 40 min	[74]
	NiO_2–MWCNTs	Chemisorption, physisorption	pH 7, 2000 mg, 10 min	[75]
	DIC-O-MWCNTs	Chemisorption	pH 5, 250 mg, 120 min	[76]
	O-MWCNTs	Chemisorption	pH 6, 200 mg, 80 min	[77]
	MWCNTs@SiO_2-NH_2	Chemisorption	pH 5, 20 mg, 60 min	[78]
	MWCNT-SH	Chemisorption	pH 5, 100 mg, 5–40 min	[79]
	SPP-MWCNTs	Chemisorption	pH 5, 10 mg, 60 min	[80]
	O-MWCNTs	Chemisorption, EI	pH 5, 30 mg, 180 & 360 min	[81]
	O-MWCNTs	Chemisorption	pH 5, 10 mg, 10 min	[82]
	CNT–steel slag	Chemisorption, IE, precipitation	pH 6.5, 20 mg, 90 min	[83]
	CoBi-LDH-Cr@CNT	Chemisorption	pH 7, 5 mg, 180 min	[84]
	MWCNT-Au/Fe_3O_4	Van der Waals, EI, IE, complexation	pH 7, 100 mg, 100 min	[85]
**As (V)**	O-MWCNTs	Chemisorption, EI	pH 4, 100 mg, 45 min	[86]

	Ce-Fe-MWCNTs	Chemisorption, EI	pH 4, 10 mg, 360 min	[87]
	Fe $(NO_3)_3$-CNT	Chemisorption, physisorption, Coprecipitation	pH 5-6, 100 mg, 120 min	[88]
	ZVI-MWCNTs	Chemisorption, complexation	pH 7, 2500 mg, 240 min	[89]
	Fe_2O_3-SWCNTs	Chemisorption	pH 4, 100 mg, 3 min	[90]
	CF-MWCNTs	Chemisorption	pH 6.5, 2000 mg, 240 min	[91]
	Zn-BDC@CT-CNT	Chemisorption	pH 4.0,10 mg, 20 min	[92]
	MWCNTs-KOH@NiNPs	EI, surface adsorption, IE, pore diffusion	pH 5, 40 mg, 30 min	[61]
	Co-Fe-N-CNTs	Coprecipitation	pH 4.5100 mg, 30 min	[93]
	Ce-Fe-MWCNTs	Chemisorption, complexation	pH 7.5, 10 mg, 360 min	[87]
	ZVI-MWCNTs	Chemisorption, complexation	pH 7, 2500 mg, 240 min	[89]
	CNTs-DES	Chemisorption	pH 2.7, 10 mg, 30 min	[62]
**As (III)**	MA-MWCNTs	Chemisorption, liquid film diffusion	pH 6.0, 2000 mg, 60 min	[94]
	$ZrO(OH)_2$-CNTs	Chemisorption, EI	pH 7, 150 mg, 360 min	[95]
	MWCNTs-3-AP	Chemisorption, physisorption	pH 7.7, 20 mg, 120 min	[96]
	CNTs–PAMAM–Ag	Chemisorption, IPD, boundary layer effect	pH 8100 mg, 15 min	[97]
	S-MWCNTs	Physisorption, EI	pH 6, 25 mg, 360 min	[98]
	SWCNTs-OH-RGO	π–π interactions	pH 6.8, < 50 mg, 180 min	[99]
	DTCA-MWCNTs	Chemisorption, physisorption	pH 6, 5.0 mg, 120 min	[100]
	PHB-CNTs	IE, EI	pH 5.65, 20 mg,10 min	[101]
**Cu (II)**	NN-mSiO_2-MWCNTs	Chemisorption	pH 6.2, 20 mg, 30 min	[102]
	Ag-MWCNTs	Chemisorption, IE	pH 6, 50 mg, 100 min	[103]
	CS-MWCNTs	Chemisorption, physisorption	pH 5.5, 50 mg, 90 min	[104]
	As-produced MWCNT	Chemisorption, EI	pH 5.5, 20 mg 120 min	[103]
	O-MWCNTs	Chemisorption, EI	pH 5.5, 1000 mg 120 min	[66]

	O-MWCNTs	Chemisorption, EI	pH 6, 30 mg, 75 min	[105]
	O-MWCNTs	EI	pH 5, 5 mg, 30 min	[106]
	MWCNT-TA	Charge interaction	pH 6, 10 mg, 240 min	[107]
	ACh-CNTs	Chemisorption, surface diffusion	pH 7, 50 mg, 300 min	[108]
	Acidified-MWCNTs	Chemisorption, IE, complexation	pH 9, 50 mg, 600 min	[73]
	M-MWCNTs	Chemisorption, surface diffusion, EI	pH 7, 100 mg, 30 min	[109,110]
	TR-CNTs	EI, pore filling, H-bonding, complexation.	pH 6, 400 mg, 60 min	[111]
	PAAm/FMWCNTs	Chemisorption, complexation	pH 5, 1000 mg 90 min	[112]
	THF-CNTs	Chemisorption	pH 5, 200 mg 30 min	[113]
	DA-MALI-CNTs	Chemisorption, chelation	pH 7, 10 mg, 60 min	[114]
	O-MWCNTs	EI, π–π interaction	pH 6, 240 min	[115]
	Double-O-MWCNTs	Chemisorption	pH 7, 20 mg, 840 min	[116]
	CNF/MWCNTs/SnO_2	Chemisorption, EI	pH 6,50 mg, 30 min	[117]
	MWCNT-PEI	Chemisorption	pH 7,10 mg, 180 min	[118]
	MWCNTs-PEG-PVA	Chemisorption, physisorption	pH 6, 20 mg, 80 min	[119]
	MWCNT-RAFT	Complexation, EI	pH 7, 1 mg, 180 min	[120]
**Cd (II)**	Al_2O_3-MWCNTs	EI, physisorption, surface precipitation, surface complexation	pH 7, 50 mg, 240 min	[121]
	Acid modified CNTs	EI	pH 7, 50 mg, 240 min	[122]
	Ag-MWCNTs	Chemisorption, IE	pH 7, 50 mg, 100 min	[103]
Empty Cell	O-MWCNT	Chemisorption, EI	pH 5.5, 1000 mg 120 min	[66]
	Raw CNTs	EI	pH 4, 75 mg, 240 min	[123]
	PP-MWCNTs	Chemisorption, physisorption	pH 6, 60 mg, 60 min	[69]
	MWCNTs-COOH	Chemisorption, EI	pH 6, 60 mg, 60 min	[69]

Cr (VI)	Acidified- MWCNTs	Chemisorption, IE, complexation	pH 9, 50 mg, 600 min	[73]
	M-MWCNTs-8-AQ	Chemisorption	pH 6.4, 25 mg, 5 min	[64]
	O-CNTs	Chemisorption, surface diffusion	pH 4, 40 mg, 300 min	[124]
	Pure CNTs	Chemisorption, physisorption	pH 10,500 mg, 500 min	[125]
	NiONPs-MWCNTs	Chemisorption, EI	pH 7-8, 200 mg, 20 min	[126]
	Modified MWCNTs	Chemisorption, physisorption	pH 5, 1000 mg, 1440 min	[127,128]
	MWCNTs-KOH@NiNPs	EI, surface adsorption, IE, pore diffusion	pH 5, 40 mg, 30 min	[61]
	PA-CNT	EI, complexation, π–metal interactions, FGI	pH 7, 30 mg, -	[129]
	MWCNTs	Chemisorption, physisorption, EI	pH 4, 100 mg, 720 min	[130]
	NCs	Chemisorption, physisorption, EI	pH 2, 2500 mg, 240 min	[131]
	O-MWCNT	Chemisorption, EI	pH 5.5, 1000 mg 120 min	[66]
	IL-oxi-MWCNTs	EI, cation/anion-interaction	pH 2.5-4.0, 150 mg, 40 min	[132]
	Raw CNTs	EI	pH 4, 75 mg, 240 min	[133]
	pTSA-Pani@GO-CNT	EI, π–π interaction	pH 2, 200 mg, 500 min	[134]
	M-MWCNTs	Chemisorption	pH 3, 100 mg, 700 min	[135]
	SM-MWCNTs	IPD	pH 1, 5 mg, 60 min	[136]
	EDTA/H_2SO_4- MWCNTs	Chemisorption, IPD	pH 3, 60 mg, 150 min	[137]
	CC-CNTs	Chemisorption, physisorption	pH 3, 10 mg, 660 min	[138]
	Fe_3C-CNTs	EI, complexation	pH 5.4,10 mg, 1440 min	[139]
	PA-CNTs	Redox, complexation, EI	pH 4, 400 mg, 3 min	[139]
	CaO_2-CNTs	Chemisorption	pH 3, 200 mg, 120 min	[140]
	MWCNTs-Ag0 /PVAc	Chemisorption	pH 3, 40 mg, 60 min	[61]
	AOMW-MWCNTs	EI, chemisorption, IE	pH 2, 25 mg, 120 min	[141]
	mZVI-CNTs	EI, H-bonding, reduction, precipitation	pH 5,1000 mg, 2100 min	[142]

	Ch/MWCNTs-COOH	Reduction, EI	pH 2, 50 mg, 30 min	[143]
	Ni@N-CNT	Reduction, EI	100 mg, 15 min	[144]
	CNTs	Chemisorption, EI	pH 10, 900 mg, 120 min	[145]
	MWCNTs	EI	pH 10, 90 mg 120 min	[146]
	O-MWCNTs	EI	pH 5, 5 mg, 30 min	[106]
	Fe_3O_4/O-MWCNTs	Chemisorption, complexation, EI	pH 6, 1000 mg, 360 min	[70]
**Zn (II)**	MWCNTs	Chemisorption	pH 5,60 mg, 360 min	[63]
	MWCNTs	Chemisorption, IE, EI	pH 10, 50 mg, 60	[145]
	f-MWCNT	Surface complexation	pH 7, 20 mg, 60 min	[147]
	Ar/O_2-CNTs	Surface complexation	pH 7, 29400 mg, 60 min	[148]
	O-CNTs	Chemisorption, EI	pH 6, 50 mg, 100 min	[63]
	As-produced MWCNT	Chemisorption, EI	pH 5.5, 20 mg, 120 min	[66]
	O-MWCNTs	Chemisorption, EI	pH 5.5, 1000 mg, 120 min	[66]
	PP-MWCNTs	Chemisorption, physisorption	pH 6, 60 mg, 60 min	[69]
**Ni (II)**	PAMAM/CNTs	Chemisorption, physisorption, EI, chelating	pH 7, 30 mg, 15 min	[149]
	Acidified MWCNTS	Chemisorption, complexation, IE	pH 9, 500 mg, 600 min	[73]
	MWCNTs	Chemisorption, IPD, EI	pH 8, 5000 mg, 300 min	[150]
	MWCNTs	Chemisorption	pH 5, 30 mg, 60 min	[151]
**Co (II)**	MWCNTs-HAP	Surface complexation	pH 6, 60 mg, 20 min	[151]
	PAMAM/CNTs	Chemisorption, physisorption, EI, chelating	pH 7, 30 mg, 15 min	[148]
	NaAlg-CNTs	EI	pH 6.8, 100 mg, 540 min	[152]
Empty Cell	SMMWCNTs	EI, complexation	pH 6, 4.5 min, 300 mg	[153]
	MWNCTs	EI	pH 10, 38.6 min, 1.57 mg	[154]

* Optimized conditions; CT = contact time; AD = absorbent dose expressed in mg/L; ATPPBr = allyl triphenyl phosphonium bromide; TBABr = tetra n-butyl ammonium bromide; PAAA = poly-(allyl acetoacetate); GO = graphene oxide; ChC = chitosan-coated; 3-AP = 3-aminopyrazole; SM = super

magnetic; Gly = glycerol; AA = amido amine; PVAc = polyvinyl acetate; DES = deep eutectic solvent; T2AEA = tris-(2-aminoethyl)-amine; M-MWCNT = magnetic MWCNT; 8-AQ = 8-aminoquiline; PP = poly pyrrole; O-MWCNT = oxidised MWCNT; PAMAM = poly amido amine; DIC = di-iodo-carbene; SPP = seleno phosphoryl; ZVI = zero valent iron; CF = carbon foam; MA = microwave-assisted; RGO reduced graphene oxide; DTCA = di thiocarbamate; PHB = poly-hydroxyl butyrate; Ach = acid chitosan; IE – ionic exchange; EI = electrostatic interactions; ED = external diffusion; SASBI = soft acid soft base interactions; IPD = intraparticle diffusion; FGI = functional groups interactions.

Table S5. CNTs as adsorbent materials to remove heavy metals in wastewater treatment.

Adsorbent	Synthesis Method	Xenobiotic	Operating parameters	Removal efficiency/Adsorption capacity	Refs.
MI MWCNT	N.A.	Cu (II)	CT = 35 min, DA = 100 mg, pH = 5.5, AS = 160 r/min IM= Freundlich and Langmuir, KM = PSO	99.9%	[155]
MWCNT	TMW CVD	Pb (II)	pH = 5, IC Pb (II) = 10 mg/L, AS = 2.8 r/*sec* DA = 100 mg, CT = 22.5 min IM = Langmuir and Freundlich, KM = PSO	99.9%	[156]
F-MWCNT	N.A.	Cu (II)	CT = 1 h, DA = 10 mg/L, IC = 20 mg, pH = 3 IM = Langmuir, KM = PSO	93%	[157]
CNT/WCPO/Fe	Thermal CVD	Zn (II), Cu (II) Fe (II)	DA = 1800 mg/L, pH = 7, IC heavy metal = 100 mg/L IM = Langmuir for Cu (II)	99.2-99.9%	[158]
MoS_2+TF-MWCNT	TMW CVD	Pb (II), Cd (II)	IM = Freundlich, KM = PSO, DA = 2 mg/mL, pH = 6 CT = 1 h	Cd (II) = 66.6 mg/g, Pb (II) = 90 mg/g	[159]
MWCNT-COOH MWCNT-OH	Catalytic CVD	Cr (VI)	IC Cr (VI) = 5–60 mg/L, pH = 5, DA = 25 mg KM = IPDM and PSO	8.09 mg/g (MWCNT-COOH) 7.85 mg/g (MWCNT-OH)	[160]

N.A. = Not available; AD = adsorbent dose; F = functionalized; TF = thiol-functionalized; CVD = chemical vapor deposition; TMW CVD = tubular microwave CVD; AS = agitation speed; IM = isotherm model; KM = kinetic model; IC = initial concentration; CT = contact time; PSO = pseudo second order kinetic; IPDM = intraparticle diffusion kinetic model.

Table S6. Applications of CNTs for the removal of pesticides, pharmaceuticals and some other emerging organic pollutants by adsorption.

Target xenobiotic	Adsorbent	MAC (mg/g) or RE (%)	Optimized condition	Ref.
1PBA, DDB	SWCNTs	50% (1PBA), 28% (DDB)	↑CLD, TH = 50 μm; Xenobiotic = 15 μg/mL	[161]
Oxytetracycline (OXY)	MWCNTs	450 mg/g	pH 7, 1560 min,100 mg	[162,163]
Ibrupofen	MWCNTs	9 mg/g	pH 4, 2.4 mg	[164]
	MWCNTs	12 mg/g	pH 4, 30 min, 50 mg	[165]
Tetracycline	MWCNTs	42 mg/g	pH 10, 2.4 mg	[164]
Empty Cell	CNTs–C@Fe–CS	104 mg/g	pH 10, 1440 min, 100 mg	[106]
Empty Cell	Fe_2O_3@OCNT	96%	pH 6, 0.13 min	[166]
Sulfamethazine	MWCNTs	34 mg/g	pH 5, 1440, 320 mg	[167]
Basic Red 46	SWCNT-COOH	49 mg/g	pH 9,10 min, 50 mg	[168]
Carbamazepine	GCNTs	87 mg/g	pH 7, 4320 min,150 mg	[169]
Diclofenac sodium	GCNTs	65 mg/g	pH 4, 4320 min,150 mg	[169]
Fulvic acids	H_2O_2-MWCNT	322 mg/g	30 min, 2 mg	[170]
Atrazine	MCNTs	58 mg/g	pH 7, 180 min, 500 mg	[171]
Azorubine	Fe_2O_3-MWCNT	> 95%	pH 5, 20 min, 130 mg	[172]
Diquat dibromide	MWCNTs	58 mg/g	pH 6.5, 300 min, 10 mg	[173]
Toluene	(MWCNTs)-SiO_2	50 mg/g	pH (2-3), 30 min, 1000 mg	[174]
Cymoxanil	CNT-COOH/MnO_2/Fe_3O_4	11 mg/g	pH 10, 5 min, 5 mg	[175]
Pirimicarb	CNT/Fe_3O_4	95%	pH 6.72, 47.56 min, 1070 mg	[176]
Malathion	F-MWCNTs	98%	pH 12, 50 min, 5 mg	[177]

1-PBA = 1-pyrene butyric acid; DDB = diquat dibromide; MAC = Maximum adsorption capacity; RE% = removal efficiency percentage; F-MWCNT = functionalized MWCNT; GCNT = granular carbon nanotube; MCNT = magnetic carbon nanotube; optimized conditions refer in the order to values of pH, contact time and adsorbent amount; CLD = cross-linked degree; TH = thickness; ↑ high, higher; @ = activated.

Table S7. CNTs as adsorbent materials to remove dyes from wastewater (WW).

Adsorbent	Synthesis Method	Xenobiotic	Operating parameters	Removal efficiency/Adsorption capacity	Refs.
SWCNT	ADSC/Crosslinking	MB	↑Cross-linking degree, thickness = 50 μm<br>Xenobiotic = 15 μg/mL	83% (MB)	[161]
MWCNT	Commercial	CR, PR dyes	pH = 6, T = 25° C, dye = 200 mg/L, dose = 1 g/L	PR = 68 mg/g, CR = 256 mg/g	[163]
MWCNT	Commercial	CR	CT = 1 h, pH = 11, T = Endothermic<br>IC = 200 ppm, MWCNT dose = 50 mg	92%	[178]
MWCNT	Catalytic CVD	Yellow 81, Red 159<br>Blue 116	IC dye = 100 mg/L, CNT = 60 mg, PSO<br>IM: Blue 16 = Temkin<br>IM: Red 159 = Langmuir/Freundlich<br>IM: Yellow 81 = Freundlich/Temkin	33 mg/g	[179]
MWCNT	Catalytic CVD	MB, MO	CT = 1 h, catalyst dose = 20 mg, IC dye = 10 mg/L<br>T = 298 K, pH = 6, KM = PSO	MB = 7 mg/g, MO = 5.5 mg/g	[180]
Chitosjan+SiO_2+MWCNT	Gelation method	RB19, DB 71	pH = 6.8 (DB71), pH = 2 (RB 19), IM = Langmuir<br>KM = PSO, R^2 = 0.996 (DB71), 0.998 (RB19)	DB71 = 61.4 mg/g, RB19 = 97.1 mg/g	[181]
CNTs.	N.A.	DR 1, AB 113<br>MB, MO	KM = PSO, IM= Langmuir, IC dye = 20 mg/L, T= 25°C<br>Dose (mg/g) = 19.4 (DR1 and MB<br>18.4 (AB113), 19 for (MO)<br>pH = 8 (MB), 7 (AB113), 6 (MO), 7 (DR1)<br>CT = 15 min (MB, MO, AB113), 10 min (DR1)	DR1 = 500 mg/g, MB = 91 mg/g<br>MO = 96 mg/g, AB113 = 172 mg/g	[182]
Fe_3O_4/AA/IPAA/MWCNTs	N.A.	MB, RhB, CV	KM = PSO, IM = Langmuir, IC dye = 50 mg/L<br>AD = 50 mg, pH = 8, T = 25°C	CV = 287 mg/g, MB = 302 mg/g<br>RhB = 231 mg/g	[183]

Gel/F_2O_3/MWCNT	Co-P/EM	MB, DR 80	IM = Freundlich, KM = PSO, T = 65°C, time = 6 h<br>IC = 500 mg/g	DR81 = 96%, MB = 76%	[184]

N.A. = Not available; gel = Gelatine; AA = acrylic acid; IPAA = isopropyl acryl amide; MI = microwave induced; AD = adsorbent dose; ADSC = aryl diazonium salt chemistry; F = functionalized; TF = thiol-functionalized; CVD = chemical vapor deposition; TMW CVD = tubular microwave CVD; Co-P/EM = co-precipitation/emulsification; AS = agitation speed; IM = isotherm model; IC = initial concentration; CT = contact time; T = temperature; ⬆ high, higher; PSO = pseudo second order kinetic; IPDM = intraparticle diffusion kinetic model; PR = ponceau 4R dyes; RB19 = reactive Blue 19; DR71 = direct Blue 71; DR1 = disperse red 1; AB113 = acid blue 113; CV = crystal violet; DR80 = direct red 80; WCPO = waste cooking palm oil; KM = kinetic model.

Table S8. Application of CNTs for the photocatalytic oxidative degradation of organic contaminants in WW.

Photocatalyst	Methods	Light source	Xenobiotic	Operating parameters	Removal efficiency	Refs.
MWCNT/TiO_2	Sol-gel	Vis	MB	MWCNT/TiO_2 molar ratio (wt) 0.05/1<br>LI = 15 W, CT = 400 C, time = 180 min, XC = 1×10^{-5} M	TiO_2 only 22%<br>MWCNT+TiO_2 70%	[185]
MWCNT/C_3N_4	Hydrothermal	Vis	RhB, MB and MO	LI = 300 W, Λ ≥ 400 nm, CD = 5 mg/mL<br>IT = 1.5 h (MB), 3 h (MO and RhB)	MB 67%, MO 90%, RhB 85%	[186]
CNT/P-TiO_2	Hydrothermal	UV and Vis	MO	Mass ratio CNT/P-TiO_2 5/100, LI UV = 250 W<br>LI Vis = 400 W, IT = 80 min	~100%	[187]
CNT/g-C_3N_4/$BiVO_4$	Wet impregnation	Simulated sunlight	Phenolic compounds	IR = 2 h, PFO, Temkin model, PC = 10 mg/L, LI = 500 W	81%	[188]
CNT/TiO_2/Al_2O_3	Hydrothermal	UV	Metamifop herbicide	CD = 10 mg, XC = 5 ppm	85%	[189]
CNT/TiO_2/PAN	Electro-spinning	UV	Phenol	pH = 5, IT = 7 min, LI = 100 W, CD = 20 mg<br>PC = 10 ppm, R&R = 3 cycles	99%	[190]
CuO/CNTs	Chemical method	UV	DR and RR	LI = 9 W, FO, RR 0.0105 (RR120), 0.0137 (DR31) min^{-1}<br>CD = 5 mg	RR 87%, DR 89%	[191]
Ti^{3+}/TiO_2/SWCNT	CP + calcination	Vis	MB	IT = 25 min, RR = 0.0083 min^{-1}	83%	[192]

MWCNT/TiO_2/SiO_2	Sol-gel	UV	CBZ and BPA	PFO<br>RC = 0.0131–0.0743 (CBZ), 0.0827–0.1751 (PBA) min^{-1}<br>IT = 20 min, CC = 500 mg/L	50%	[193]
MWCNT/Ag-ZnO	Precipitation	Vis	CR	CC = 150 mg/L, LI = 40 W, pH = 6, XC = 15 mg/L<br>FO, RC = 0.0023 min^{-1}	99%	[194]

CP = Chemical precipitation; PAN = polyacrylonitrile; MB = methylene blue; RhB = rhodamine B; MO = methylene orange; DR = direct red; RR = reactive red CBZ = carbamazepine, BPA = Bisphenol A; CR = Congo red (CR); Vis = visible; LI = light intensity; CT = calcination temperature; XC = xenobiotic concentration; CD = catalyst dose; CC = catalyst concentration; IT = irradiance time; PFO = pseudo first order kinetic model; FO = first order kinetic model; PC = phenol concentration; R&R = recycle and reuse; RR = reaction rate; RC = rate constant.

Table S9. CNTs-based membranes and filtration systems for wastewater treatment.

Membrane/Filter	Material	Methods	Membrane performance	Refs.
VACNT	CNT+PES	CVD	UF, WTS = 100 L/m^2. h at 60 Psi, 3 times faster than RO CNT+PES<br>10 times faster than PES membrane	[195]
VACNT	CNT+Epoxy	WAT CVD	3 times ⬆ WF than UF, 2 log ⬇ bacterial with VCNT than with UF<br>PFR (after 600 min) = 67% for VCNT and 55% for UF	[196]
VACNT	CNT+SSM	Thermal CVD	Separate water layer and diesel and even SSE<br>⬆Hydrophobicity and oleophobicity	[197]
SWCNT	CNT+PVF	Vacuum filtration	⬇5×10^5 *E coli*, CI = 79% after 20 min CT, MA *E. Coli* = 6%, damaged *E coli* membrane	[198]
VACNT	CNT+PTE+Si *	WA CVD	Millimeter thick UFM, WP = 30000$Lm^{-2}h^{-1}bar^{-1}$, ⬇bacterial growth, ⬇biofilm formation	[199]
VACNT	MWCNT+Fe+Al_2O_3+Si *	Thermal CVD	PIF/TR = 24% (UFM), 69% (VCNTM), PRFR = 38% (UFM), 5% (VCNTM) in BSA<br>⬆ Removal 71% to 90% after surface modification by GP of MAA	[200]
VA DWCNT	DWCNT+Si wafer *	CVD	NaCl rejection = 41.4%, WF = 1.31×10^{-3} to 62.7×10^{-3} L cm^{-2} day^{-1} Mpa^{-1}	[201]
CNT BP	CNT	CVD, UV/OT/AS	SR ≥95%, LS ⬆ by 50%	[202]
VACNT	MCNT+PDMS+Si *	CVD	SR = 96.5% at OP of 2 bar	[203]
GO-VACNT<br>PAC-VACNT	CNT+Epoxy	N.A.	SR = 45% (GO-VCNT), 65% (PAC-VCNT) at OP of 15.5 bar	[204]
PA/outer-wall VACNT	CNT+Epoxy	IP	ROM, Flux = 128.6 $Lm^{-2}h^{-1}$, SR = 98.3% at OP of 15.5 bar	[205]

N.A. = Not available; PES = poly ether sulfone; PVF = poly vinylidene fluoride; PTE = poly tetrafluoro ethylene; DWCNTs = double-wall CNT; PDMS = polydimethylsiloxane; SSM = stainless steel mesh; * substrate; VA = vertical aligned; VACNTs = vertical aligned CNTs; GO-VACNT = graphene oxide coated VACNT; PA polyamide; PAC-VACNT = polyamide coated VACNT; CNT BP = CNT bucky paper; IP = interfacial polymerization; CVD = chemical vapor deposition; WAT CVD = water assisted thermal CVD; WA CVD = water assisted CVD; OT = ozone treatment; AS = alkoxy silanation; UF = used for ultrafiltration; WTS = water transportation speed; RO CNT = random oriented CNT; WF = water flux; PFR = permeate flux reduction; SSE = surfactant stabilized emulsions; CI = cell inactivation, CT = contact time; MB = metabolically active; WP = water permeability; PIF/TR = proportion of irreversible fouling to total resistance; PRFR = proportion of reversible fouling resistance; BSA = bovine serum albumin; RI = removal improvement;

GP = graft-polymerization; MAA = methacrylic acid, WF = water flux; LS = life span; SR = salt rejection; OP = operational pressure; PO-VCNT = ; ROM = reverse osmosis membrane; UFM = ultrafiltration membrane; VCNTM = vertical CNT membrane; ↑ = high, higher, improve, improved.

Table S10. Applications of CNTs-based filtering membranes for water desalination.

Membrane	PM	OOP	Effect of CNTs	RE	Refs
MWCNT+CA	PI	1% CNT+CAM, FS = $MgSO_4$ WF =69.5 $Lm^{-2}h^{-1}$	↑Permeability	90.6% ($MgSO_4$), 83.3% (Na_2SO_4), 44.6% (NaCl)	[206]
CNT+PES	PI	CNT C = 0.1 wt%, WF =38.91$Lm^{-2}h^{-1}$ P = 4 bar; FS = 200 ppm Na_2SO_4, $MgSO_4$, NaCl	↑SR and WF	72.2% ($MgSO_4$), 87.3% (Na_2SO_4), 24.7% (NaCl)	[207]
PS20/BTCT/MPD/MWCNT	IP	FS = 2000 ppm NaCl, pH = 6–7, T= 25°C P = 225psi, WF = 43 $Lm^{-2}h^{-1}$	↑WP, WF, SH	99%	[208]
MWCNT-TN/PA	IP	P = 15 bar, WF = 0.74 $Lm^{-2}h^{-1}$ FS = 2000 ppm NaCl	↑CA, SC, SRGH	98% (NaCl), 98.1 (Na_2SO_4)	[209]
MVF PES/SPS/O-MWCNT	IP	FS = brackish waters; SC =2000 ppm, P= 3 bar WF = 30.2 $Lm^{-2}h^{-1}bar^{-1}$	↑SR, WP	SS (NaCl/Na_2SO_4) = 25	[210]
CNT/PA	E-A IP	FS = 1 g/L NaCl, P = 4 bar, WF = 96.8 $Lm^{-2}h^{-1}$	↑WF	89.6%	[211]
SWCNT/PA	Brush painting	FS = 1 g/L Na_2SO_4, P = 6 bar, WF = 40 $Lm^{-2}h^{-1}$	↑WP	96.5%	[212]
RGO/CNT	ED/CR	FS = 0.1 M NaCl, P = 1 bar, WF = 40.4±3.7 $Lm^{-2}h^{-1}$	↑WF	94±1.9%	[213]
PPC/MWCNT	IP	FS = 2000 mg/L Na_2SO_4, P = 1 MPa, = 65.7 $Lm^{-2}h^{-1}$	↑WP, SR	97.6 %	[214]

PM = Preparation methods; OOP = operation parameters; RE = removal efficiency; CA = cellulose acetate (CA); PES = poly ether sulfone; PS-20 = poly sulfone; BTCT = 1,3,5-benzenetricarbonyl trichloride; MPD = m-phenylenediamine; TN = titania nanotube; PAMVF = polyamide macro void-free PES; SPS = sulfonated poly sulfone; O-MWCNTs = oxidized MWCNT; RGO = reduced graphene oxide; PPC = poly pyrrole coated; IP = interfacial polymerization; E-A = electrospray-assisted; PI = phase inversion; ED = electrophoretic deposition; CR = chemical reduction; CAM = CA membrane; FS = feed system or feed solution; WF = water flux; CNT C = CNT concentration; P = pressure; T = temperature; SC = salt concentration; SS = salt selectivity; SR = salt rejection; WP = water permeability; SH = surface hydrophilicity; CA = contact angle; SC = surface charge; SRGH = surface roughness.

Table S11. Main developed methods for CNTs regeneration.

Adsorbents	Xenobiotic	Regeneration mode	Cycles	Results after last cycle	Ref.
O-MWCNT	Cadmium	Physical	-	At pH 1.5, 93% of Cd^{2+} RE	[215]
CNTs/ Fe_3O_4	Biphenol A	Chemical	5	No valuable loss in AC	[216]
Chitosan-MWCNTs	Congo Red dye	Chemical (0.01 M NaOH)	3	RR 71% after 3 cycles	[217]
O-MWCNT	BTX	Chemical	5	Only 5.15%, 0.97%, 1.05% (BTX) RE	[218]
MWCNTs- Cu-$NiFe_2O_4$	Oxytetracycline HCl	Chemical (0.1 M NaOH)	3	RE = 45.6%	[166]
Magnetic MWCNTs	Gatifloxacin	Chemical	5	Less than 7.8% recovery loss	[219]
MWCNTs	CO_2	TT and VI	20	Only 3% attrition after 20 cycles	[220]
MWCNTs	Anticancer drugs	TT	5	No negative influence on the sorption level	[221]
Magnetic MWCNTs	Atrazine	Ozone-assisted	10	Retained 85-93% of its AC	[171]
NaOCl-MWCNTs	Molybdenum	Chemical (0.15 M HNO_3)	10	89.5% RE with 3.6 wt% NaOCl	[222]
MWCNTs	AAP, ibuprofen, TCS	Chemical, ultrasonic and thermal	4-5	Sonication ↑ RE%, 100% RE after TT (380 °C)	[223]
MWCNTs	Tetracycline	Microwave-UV system	5	AC of regenerated CNT = 100%	[224]
MWCNTs	Reactive red 3BS	Microwave irradiation	4	RE 92.8% but ↓ AC	[225]
CNTs	Reactive Black dye	Electrochemical	2	RE 86.5% (MWCNTs) and 77.3% (SWCNTs)	[226]
Magnetic MWCNTs	p-Nitrophenol	Microwave-assisted	6	AC = 19.7 mg/g at 850 W, RE = 106%	[227]

AAP = Acetaminophen; TCS = triclosan; VI = Vacuum interactions; AC = absorption capacity; RE = regeneration efficiency; TT = thermal treatment; BTX = benzene, toluene and xylenes; RR = regeneration rate; UV = ultraviolet.

References

1. Iijima, S. Helical Microtubules of Graphitic Carbon. *Nature* **1991**, *354*, 56–58, doi:10.1038/354056a0.
2. Ebbesen, T.W.; Ajayan, P.M. Large-Scale Synthesis of Carbon Nanotubes. *Nature* **1992**, *358*, 220–222, doi:10.1038/358220a0.
3. Eatemadi, A.; Daraee, H.; Karimkhanloo, H.; Kouhi, M.; Zarghami, N.; Akbarzadeh, A.; Abasi, M.; Hanifehpour, Y.; Joo, S.W. Carbon Nanotubes: Properties, Synthesis, Purification, and Medical Applications. *Nanoscale Res Lett* **2014**, *9*, 393, doi:10.1186/1556-276X-9-393.
4. Alfei, S.; Schito, G.C. Antimicrobial Nanotubes Between Promising Outcomes, Un-Anticipated Toxicities, Strategies to Limit Them and Regulatory Issues: A Review. *Preprint* **2025**, 2025031472.
5. Alfei, S.; Schito, G.C. Nanotubes: Carbon-Based Fibers and Bacterial Nano-Conduits Both Arousing a Global Interest and Conflicting Opinions. *Fibers* **2022**, *10*, 75, doi:10.3390/fib10090075.
6. Guo, T.; Nikolaev, P.; Rinzler, A.G.; Tomanek, D.; Colbert, D.T.; Smalley, R.E. Self-Assembly of Tubular Fullerenes. *J Phys Chem* **1995**, *99*, 10694–10697, doi:10.1021/j100027a002.
7. Guo, T.; Nikolaev, P.; Thess, A.; Colbert, D.T.; Smalley, R.E. Catalytic Growth of Single-Walled Nanotubes by Laser Vaporization. *Chem Phys Lett* **1995**, *243*, 49–54, doi:10.1016/0009-2614(95)00825-O.
8. Kumar, M.; Ando, Y. Chemical Vapor Deposition of Carbon Nanotubes: A Review on Growth Mechanism and Mass Production. *J Nanosci Nanotechnol* **2010**, *10*, 3739–3758, doi:10.1166/jnn.2010.2939.
9. Neupane, S.; Lastres, M.; Chiarella, M.; Li, W.; Su, Q.; Du, G. Synthesis and Field Emission Properties of Vertically Aligned Carbon Nanotube Arrays on Copper. *Carbon N Y* **2012**, *50*, 2641–2650, doi:10.1016/j.carbon.2012.02.024.
10. Inami, N.; Mohamed, M.A.; Shikoh, E.; Fujiwara, A. Synthesis-Condition Dependence of Carbon Nanotube Growth by Alcohol Catalytic Chemical Vapor Deposition Method. *Sci Technol Adv Mater* **2007**, *8*, 292–295, doi:10.1016/j.stam.2007.02.009.
11. Ishigami, N.; Ago, H.; Imamoto, K.; Tsuji, M.; Iakoubovskii, K.; Minami, N. Crystal Plane Dependent Growth of Aligned Single-Walled Carbon Nanotubes on Sapphire. *J Am Chem Soc* **2008**, *130*, 9918–9924, doi:10.1021/ja8024752.
12. Naha, S.; Puri, I.K. A Model for Catalytic Growth of Carbon Nanotubes. *J Phys D Appl Phys* **2008**, *41*, 065304, doi:10.1088/0022-3727/41/6/065304.
13. Banerjee, S.; Naha, S.; Puri, I.K. Molecular Simulation of the Carbon Nanotube Growth Mode during Catalytic Synthesis. *Appl Phys Lett* **2008**, *92*, doi:10.1063/1.2945798.
14. Eftekhari, A.; Jafarkhani, P.; Moztarzadeh, F. High-Yield Synthesis of Carbon Nanotubes Using a Water-Soluble Catalyst Support in Catalytic Chemical Vapor Deposition. *Carbon N Y* **2006**, *44*, 1343–1345, doi:10.1016/j.carbon.2005.12.006.
15. Ren, Z.F.; Huang, Z.P.; Xu, J.W.; Wang, J.H.; Bush, P.; Siegal, M.P.; Provencio, P.N. Synthesis of Large Arrays of Well-Aligned Carbon Nanotubes on Glass. *Science (1979)* **1998**, *282*, 1105–1107, doi:10.1126/science.282.5391.1105.
16. Futaba, D.N.; Hata, K.; Yamada, T.; Hiraoka, T.; Hayamizu, Y.; Kakudate, Y.; Tanaike, O.; Hatori, H.; Yumura, M.; Iijima, S. Shape-Engineerable and Highly Densely Packed Single-Walled Carbon Nanotubes and Their Application as Super-Capacitor Electrodes. *Nat Mater* **2006**, *5*, 987–994, doi:10.1038/nmat1782.
17. Hata, K.; Futaba, D.N.; Mizuno, K.; Namai, T.; Yumura, M.; Iijima, S. Water-Assisted Highly Efficient Synthesis of Impurity-Free Single-Walled Carbon Nanotubes. *Science (1979)* **2004**, *306*, 1362–1364, doi:10.1126/science.1104962.
18. Smiljanic, O.; Stansfield, B.L.; Dodelet, J.-P.; Serventi, A.; Désilets, S. Gas-Phase Synthesis of SWNT by an Atmospheric Pressure Plasma Jet. *Chem Phys Lett* **2002**, *356*, 189–193, doi:10.1016/S0009-2614(02)00132-X.

19. Kim, K.S.; Cota-Sanchez, G.; Kingston, C.T.; Imris, M.; Simard, B.; Soucy, G. Large-Scale Production of Single-Walled Carbon Nanotubes by Induction Thermal Plasma. *J Phys D Appl Phys* **2007**, *40*, 2375–2387, doi:10.1088/0022-3727/40/8/S17.
20. Ren, J.; Li, F.-F.; Lau, J.; González-Urbina, L.; Licht, S. One-Pot Synthesis of Carbon Nanofibers from CO_2. *Nano Lett* **2015**, *15*, 6142–6148, doi:10.1021/acs.nanolett.5b02427.
21. Service, R.F. Conjuring Chemical Cornucopias out of Thin Air. *Science (1979)* **2015**, *349*, 1160–1160, doi:10.1126/science.349.6253.1160.
22. Yuan, L.; Saito, K.; Pan, C.; Williams, F.A.; Gordon, A.S. Nanotubes from Methane Flames. *Chem Phys Lett* **2001**, *340*, 237–241, doi:10.1016/S0009-2614(01)00435-3.
23. Yuan, L.; Saito, K.; Hu, W.; Chen, Z. Ethylene Flame Synthesis of Well-Aligned Multi-Walled Carbon Nanotubes. *Chem Phys Lett* **2001**, *346*, 23–28, doi:10.1016/S0009-2614(01)00959-9.
24. Duan, H.M.; McKinnon, J.T. Nanoclusters Produced in Flames. *J Phys Chem* **1994**, *98*, 12815–12818, doi:10.1021/j100100a001.
25. Novikov, I. V.; Krasnikov, D. V.; Lee, I.H.; Agafonova, E.E.; Serebrennikova, S.I.; Lee, Y.; Kim, S.; Nam, J.; Kondrashov, V.A.; Han, J.; et al. Aerosol CVD Carbon Nanotube Thin Films: From Synthesis to Advanced Applications: A Comprehensive Review. *Advanced Materials* **2025**, doi:10.1002/adma.202413777.
26. Khabushev, E.M.; Kolodiazhnaia, J. V.; Krasnikov, D. V.; Nasibulin, A.G. Activation of Catalyst Particles for Single-Walled Carbon Nanotube Synthesis. *Chemical Engineering Journal* **2021**, *413*, 127475, doi:10.1016/J.CEJ.2020.127475.
27. Nasibulin, A.G.; Moisala, A.; Brown, D.P.; Jiang, H.; Kauppinen, E.I. A Novel Aerosol Method for Single Walled Carbon Nanotube Synthesis. *Chem Phys Lett* **2005**, *402*, 227–232, doi:10.1016/J.CPLETT.2004.12.040.
28. Lotfy, V.F.; Fathy, N.A.; Basta, A.H. Novel Approach for Synthesizing Different Shapes of Carbon Nanotubes from Rice Straw Residue. *J Environ Chem Eng* **2018**, *6*, doi:10.1016/j.jece.2018.09.055.
29. Hamid, Z.A.; Azim, A.A.; Mouez, F.A.; Rehim, S.S.A. Challenges on Synthesis of Carbon Nanotubes from Environmentally Friendly Green Oil Using Pyrolysis Technique. *J Anal Appl Pyrolysis* **2017**, *126*, doi:10.1016/j.jaap.2017.06.005.
30. Ghosh, P.; Soga, T.; Afre, R.A.; Jimbo, T. Simplified Synthesis of Single-Walled Carbon Nanotubes from a Botanical Hydrocarbon: Turpentine Oil. *J Alloys Compd* **2008**, *462*, doi:10.1016/j.jallcom.2007.08.027.
31. Paul, S.; Samdarshi, S.K. A Green Precursor for Carbon Nanotube Synthesis. *Xinxing Tan Cailiao/New Carbon Materials* **2011**, *26*, doi:10.1016/S1872-5805(11)60068-1.
32. Kumar, R.; Singh, R.K.; Tiwari, R.S. Growth Analysis and High-Yield Synthesis of Aligned-Stacked Branched Nitrogen-Doped Carbon Nanotubes Using Sesame Oil as a Natural Botanical Hydrocarbon Precursor. *Mater Des* **2016**, *94*, doi:10.1016/j.matdes.2016.01.025.
33. Suriani, A.B.; Azira, A.A.; Nik, S.F.; Md Nor, R.; Rusop, M. Synthesis of Vertically Aligned Carbon Nanotubes Using Natural Palm Oil as Carbon Precursor. *Mater Lett* **2009**, *63*, doi:10.1016/j.matlet.2009.09.048.
34. Moo, J.G.S.; Veksha, A.; Oh, W. Da; Giannis, A.; Udayanga, W.D.C.; Lin, S.X.; Ge, L.; Lisak, G. Plastic Derived Carbon Nanotubes for Electrocatalytic Oxygen Reduction Reaction: Effects of Plastic Feedstock and Synthesis Temperature. *Electrochem commun* **2019**, *101*, doi:10.1016/j.elecom.2019.02.014.
35. Abdullayeva, S.H.; Musayeva, N.N.; Jabbarov, R.B.; Matsuda, T. Synthesis of Carbon Nanotubes from Byproducts of Oil Refiner. *World Journal of Condensed Matter Physics* **2014**, *04*, doi:10.4236/wjcmp.2014.43014.
36. Wang, J.; Shen, B.; Lan, M.; Kang, D.; Wu, C. Carbon Nanotubes (CNTs) Production from Catalytic Pyrolysis of Waste Plastics: The Influence of Catalyst and Reaction Pressure. *Catal Today* 2020, *351*.

37. Boufades, D.; Hammadou Née Mesdour, S.; Moussiden, A.; Benmebrouka, H.; Ghouti, M.; Kaddour, O. Optimization of Carbon Nanotubes Synthesis via Pyrolysis over Ni/Al2O3 Using Response Surface Methodology. *Fullerenes Nanotubes and Carbon Nanostructures* **2022**, *30*, doi:10.1080/1536383X.2021.1956475.
38. Ahmad, S.; Liao, Y.; Hussain, A.; Zhang, Q.; Ding, E.X.; Jiang, H.; Kauppinen, E.I. Systematic Investigation of the Catalyst Composition Effects on Single-Walled Carbon Nanotubes Synthesis in Floating-Catalyst CVD. *Carbon N Y* **2019**, *149*, doi:10.1016/j.carbon.2019.04.026.
39. Fathy, N.A. Carbon Nanotubes Synthesis Using Carbonization of Pretreated Rice Straw through Chemical Vapor Deposition of Camphor. *RSC Adv* **2017**, *7*, doi:10.1039/c7ra04882c.
40. Debalina, B.; Reddy, R.B.; Vinu, R. Production of Carbon Nanostructures in Biochar, Bio-Oil and Gases from Bagasse via Microwave Assisted Pyrolysis Using Fe and Co as Susceptors. *J Anal Appl Pyrolysis* **2017**, *124*, doi:10.1016/j.jaap.2017.01.018.
41. Wang, Z.; Ogata, H.; Morimoto, S.; Ortiz-Medina, J.; Fujishige, M.; Takeuchi, K.; Muramatsu, H.; Hayashi, T.; Terrones, M.; Hashimoto, Y.; et al. Nanocarbons from Rice Husk by Microwave Plasma Irradiation: From Graphene and Carbon Nanotubes to Graphenated Carbon Nanotube Hybrids. *Carbon N Y* **2015**, *94*, doi:10.1016/j.carbon.2015.07.037.
42. Hildago-Oporto, P.; Navia, R.; Hunter, R.; Coronado, G.; Gonzalez, M.E. Synthesis of Carbon Nanotubes Using Biochar as Precursor Material under Microwave Irradiation. *J Environ Manage* **2019**, *244*, doi:10.1016/j.jenvman.2019.03.082.
43. Omoriyekomwan, J.E.; Tahmasebi, A.; Zhang, J.; Yu, J. Formation of Hollow Carbon Nanofibers on Bio-Char during Microwave Pyrolysis of Palm Kernel Shell. *Energy Convers Manag* **2017**, *148*, doi:10.1016/j.enconman.2017.06.022.
44. Zhang, J.; Tahmasebi, A.; Omoriyekomwan, J.E.; Yu, J. Production of Carbon Nanotubes on Bio-Char at Low Temperature via Microwave-Assisted CVD Using Ni Catalyst. *Diam Relat Mater* **2019**, *91*, doi:10.1016/j.diamond.2018.11.012.
45. Shi, K.; Yan, J.; Lester, E.; Wu, T. Catalyst-Free Synthesis of Multiwalled Carbon Nanotubes via Microwave-Induced Processing of Biomass. *Ind Eng Chem Res* **2014**, *53*, doi:10.1021/ie503076n.
46. AlOmar, M.K.; Alsaadi, M.A.; Hayyan, M.; Akib, S.; Ibrahim, M.; Hashim, M.A. Allyl Triphenyl Phosphonium Bromide Based DES-Functionalized Carbon Nanotubes for the Removal of Mercury from Water. *Chemosphere* **2017**, *167*, doi:10.1016/j.chemosphere.2016.09.133.
47. AlOmar, M.K.; Alsaadi, M.A.; Jassam, T.M.; Akib, S.; Ali Hashim, M. Novel Deep Eutectic Solvent-Functionalized Carbon Nanotubes Adsorbent for Mercury Removal from Water. *J Colloid Interface Sci* **2017**, *497*, doi:10.1016/j.jcis.2017.03.014.
48. Singha Deb, A.K.; Dwivedi, V.; Dasgupta, K.; Musharaf Ali, S.; Shenoy, K.T. Novel Amidoamine Functionalized Multi-Walled Carbon Nanotubes for Removal of Mercury(II) Ions from Wastewater: Combined Experimental and Density Functional Theoretical Approach. *Chemical Engineering Journal* **2017**, *313*, doi:10.1016/j.cej.2016.10.126.
49. Moghaddam, H.K.; Pakizeh, M. Experimental Study on Mercury Ions Removal from Aqueous Solution by MnO2/CNTs Nanocomposite Adsorbent. *Journal of Industrial and Engineering Chemistry* **2015**, *21*, doi:10.1016/j.jiec.2014.02.028.
50. Bandaru, N.M.; Reta, N.; Dalal, H.; Ellis, A. V.; Shapter, J.; Voelcker, N.H. Enhanced Adsorption of Mercury Ions on Thiol Derivatized Single Wall Carbon Nanotubes. *J Hazard Mater* **2013**, *261*, doi:10.1016/j.jhazmat.2013.07.076.
51. Pillay, K.; Cukrowska, E.M.; Coville, N.J. Improved Uptake of Mercury by Sulphur-Containing Carbon Nanotubes. *Microchemical Journal* **2013**, *108*, doi:10.1016/j.microc.2012.10.014.

52. Gupta, A.; Vidyarthi, S.R.; Sankararamakrishnan, N. Enhanced Sorption of Mercury from Compact Fluorescent Bulbs and Contaminated Water Streams Using Functionalized Multiwalled Carbon Nanotubes. *J Hazard Mater* **2014**, *274*, doi:10.1016/j.jhazmat.2014.03.020.
53. Hadavifar, M.; Bahramifar, N.; Younesi, H.; Li, Q. Adsorption of Mercury Ions from Synthetic and Real Wastewater Aqueous Solution by Functionalized Multi-Walled Carbon Nanotube with Both Amino and Thiolated Groups. *Chemical Engineering Journal* **2014**, *237*, doi:10.1016/j.cej.2013.10.014.
54. Chen, P.H.; Hsu, C.F.; Tsai, D.D.W.; Lu, Y.M.; Huang, W.J. Adsorption of Mercury from Water by Modified Multi-Walled Carbon Nanotubes: Adsorption Behaviour and Interference Resistance by Coexisting Anions. *Environmental Technology (United Kingdom)* **2014**, *35*, doi:10.1080/09593330.2014.886627.
55. Yaghmaeian, K.; Khosravi Mashizi, R.; Nasseri, S.; Mahvi, A.H.; Alimohammadi, M.; Nazmara, S. Removal of Inorganic Mercury from Aquatic Environments by Multi-Walled Carbon Nanotubes. *J Environ Health Sci Eng* **2015**, *13*, doi:10.1186/s40201-015-0209-8.
56. Alijani, H.; Shariatinia, Z. Synthesis of High Growth Rate SWCNTs and Their Magnetite Cobalt Sulfide Nanohybrid as Super-Adsorbent for Mercury Removal. *Chemical Engineering Research and Design* **2018**, *129*, doi:10.1016/j.cherd.2017.11.014.
57. Zhang, D.; Yin, Y.; Liu, J. Removal of Hg2+ and Methylmercury in Waters by Functionalized Multi-Walled Carbon Nanotubes: Adsorption Behavior and the Impacts of Some Environmentally Relevant Factors. *Chemical Speciation and Bioavailability* **2017**, *29*, doi:10.1080/09542299.2017.1378596.
58. Alimohammady, M.; Jahangiri, M.; Kiani, F.; Tahermansouri, H. A New Modified MWCNTs with 3-Aminopyrazole as a Nanoadsorbent for Cd(II) Removal from Aqueous Solutions. *J Environ Chem Eng* **2017**, *5*, doi:10.1016/j.jece.2017.06.045.
59. Fan, L.; Zhou, A.; Zhong, L.; Zhang, Z.; Liu, Y. Selective and Effective Adsorption of Hg(II) from Aqueous Solution over Wide PH Range by Thiol Functionalized Magnetic Carbon Nanotubes. *Chemosphere* **2019**, *226*, doi:10.1016/j.chemosphere.2019.03.154.
60. Liu, H.; Ruan, W.; Zhang, Z.; Zhou, Y.; Shen, F.; Liu, J.; Yang, H. Performance and Mechanism of CuS-Modified MWCNTs on Mercury Removal: Experimental and Density Functional Theory Study. *Fuel* **2022**, *309*, doi:10.1016/j.fuel.2021.122238.
61. Egbosiuba, T.C.; Egwunyenga, M.C.; Tijani, J.O.; Mustapha, S.; Abdulkareem, A.S.; Kovo, A.S.; Krikstolaityte, V.; Veksha, A.; Wagner, M.; Lisak, G. Activated Multi-Walled Carbon Nanotubes Decorated with Zero Valent Nickel Nanoparticles for Arsenic, Cadmium and Lead Adsorption from Wastewater in a Batch and Continuous Flow Modes. *J Hazard Mater* **2022**, *423*, doi:10.1016/j.jhazmat.2021.126993.
62. AlOmar, M.K.; Alsaadi, M.A.; Hayyan, M.; Akib, S.; Hashim, M.A. Functionalization of CNTs Surface with Phosphonuim Based Deep Eutectic Solvents for Arsenic Removal from Water. *Appl Surf Sci* **2016**, *389*, doi:10.1016/j.apsusc.2016.07.079.
63. Ogunsola, S.S.; Oladipo, M.E.; Oladoye, P.O.; Kadhom, M. Carbon Nanotubes for Sustainable Environmental Remediation: A Critical and Comprehensive Review. *Nano-Structures and Nano-Objects* 2024, *37*.
64. Taghizadeh, M.; Asgharinezhad, A.A.; Samkhaniany, N.; Tadjarodi, A.; Abbaszadeh, A.; Pooladi, M. Solid Phase Extraction of Heavy Metal Ions Based on a Novel Functionalized Magnetic Multi-Walled Carbon Nanotube Composite with the Aid of Experimental Design Methodology. *Microchimica Acta* **2014**, *181*, doi:10.1007/s00604-013-1154-9.
65. Saadat, S.; Karimi-Jashni, A.; Doroodmand, M.M. Synthesis and Characterization of Novel Single-Walled Carbon Nanotubes- Doped Walnut Shell Composite and Its Adsorption Performance for Lead in Aqueous Solutions. *J Environ Chem Eng* **2014**, *2*, doi:10.1016/j.jece.2014.08.024.

66. Lasheen, M.R.; El-Sherif, I.Y.; Sabry, D.Y.; El-Wakeel, S.T.; El-Shahat, M.F. Removal of Heavy Metals from Aqueous Solution by Multiwalled Carbon Nanotubes: Equilibrium, Isotherms, and Kinetics. *Desalination Water Treat* **2015**, *53*, doi:10.1080/19443994.2013.873880.
67. Ma, X.; Yang, S.T.; Tang, H.; Liu, Y.; Wang, H. Competitive Adsorption of Heavy Metal Ions on Carbon Nanotubes and the Desorption in Simulated Biofluids. *J Colloid Interface Sci* **2015**, *448*, doi:10.1016/j.jcis.2015.02.042.
68. Ouni, L.; Mirzaei, M.; Ashtari, P.; Ramazani, A.; Rahimi, M.; Bolourinovin, F. Isocyanate Functionalized Multiwalled Carbon Nanotubes for Separation of Lead from Cyclotron Production of Thallium-201. *J Radioanal Nucl Chem* **2016**, *310*, doi:10.1007/s10967-016-4928-9.
69. Kanthapazham, R.; Ayyavu, C.; Mahendiradas, D. Removal of Pb2+, Ni2+ and Cd2+ Ions in Aqueous Media Using Functionalized MWCNT Wrapped Polypyrrole Nanocomposite. *Desalination Water Treat* **2016**, *57*, doi:10.1080/19443994.2015.1081629.
70. Jiang, L.; Li, S.; Yu, H.; Zou, Z.; Hou, X.; Shen, F.; Li, C.; Yao, X. Amino and Thiol Modified Magnetic Multi-Walled Carbon Nanotubes for the Simultaneous Removal of Lead, Zinc, and Phenol from Aqueous Solutions. *Appl Surf Sci* **2016**, *369*, doi:10.1016/j.apsusc.2016.02.067.
71. Jiang, L.; Yu, H.; Zhou, X.; Hou, X.; Zou, Z.; Li, S.; Li, C.; Yao, X. Preparation, Characterization, and Adsorption Properties of Magnetic Multi-Walled Carbon Nanotubes for Simultaneous Removal of Lead(II) and Zinc(II) from Aqueous Solutions. *Desalination Water Treat* **2016**, *57*, doi:10.1080/19443994.2015.1090924.
72. Hayati, B.; Maleki, A.; Najafi, F.; Daraei, H.; Gharibi, F.; McKay, G. Super High Removal Capacities of Heavy Metals (Pb2+ and Cu2+) Using CNT Dendrimer. *J Hazard Mater* **2017**, *336*, doi:10.1016/j.jhazmat.2017.02.059.
73. Farghali, A.A.; Abdel Tawab, H.A.; Abdel Moaty, S.A.; Khaled, R. Functionalization of Acidified Multi-Walled Carbon Nanotubes for Removal of Heavy Metals in Aqueous Solutions. *J Nanostructure Chem* **2017**, *7*, doi:10.1007/s40097-017-0227-4.
74. Elmi, F.; Hosseini, T.; Taleshi, M.S.; Taleshi, F. Kinetic and Thermodynamic Investigation into the Lead Adsorption Process from Wastewater through Magnetic Nanocomposite Fe3O4/CNT. *Nanotechnology for Environmental Engineering* **2017**, *2*, doi:10.1007/s41204-017-0023-x.
75. Navaei Diva, T.; Zare, K.; Taleshi, F.; Yousefi, M. Synthesis, Characterization, and Application of Nickel Oxide/CNT Nanocomposites to Remove Pb2+ from Aqueous Solution. *J Nanostructure Chem* **2017**, *7*, doi:10.1007/s40097-017-0239-0.
76. Yang, J.Y.; Jiang, X.Y.; Jiao, F.P.; Yu, J.G.; Chen, X.Q. Fabrication of Diiodocarbene Functionalized Oxidized Multi-Walled Carbon Nanotube and Its Aqueous Adsorption Performance toward Pb(II). *Environ Earth Sci* **2017**, *76*, doi:10.1007/s12665-017-7042-6.
77. Quyen, N.D.V.; Tuyen, T.N.; Khieu, D.Q.; van Minh Hai, H.; Tin, D.X.; Lan, P.T.N.; Kiyoshi, I. Lead Ions Removal from Aqueous Solution Using Modified Carbon Nanotubes. *Bulletin of Materials Science* **2018**, *41*, doi:10.1007/s12034-017-1541-7.
78. Yang, K.; Lou, Z.; Fu, R.; Zhou, J.; Xu, J.; Baig, S.A.; Xu, X. Multiwalled Carbon Nanotubes Incorporated with or without Amino Groups for Aqueous Pb(II) Removal: Comparison and Mechanism Study. *J Mol Liq* **2018**, *260*, doi:10.1016/j.molliq.2018.03.082.
79. Ouni, L.; Ramazani, A.; Taghavi Fardood, S. An Overview of Carbon Nanotubes Role in Heavy Metals Removal from Wastewater. *Front Chem Sci Eng* 2019, *13*.
80. Kończyk, J.; Żarska, S.; Ciesielski, W. Adsorptive Removal of Pb(II) Ions from Aqueous Solutions by Multi-Walled Carbon Nanotubes Functionalised by Selenophosphoryl Groups: Kinetic, Mechanism, and Thermodynamic Studies. *Colloids Surf A Physicochem Eng Asp* **2019**, *575*, doi:10.1016/j.colsurfa.2019.04.058.

81. Solic, M.; Maletic, S.; Isakovski, M.K.; Nikic, J.; Watson, M.; Konya, Z.; Roncevic, S.D.S. Removing Low Levels of Cd(II) and Pb(II) by Adsorption on Two Types of Oxidized Multiwalled Carbon Nanotubes. *J Environ Chem Eng* **2021**, *9*, doi:10.1016/j.jece.2021.105402.
82. Fu, Q.; Lou, J.; Shi, D.; Zhou, S.; Hu, J.; Wang, Q.; Huang, W.; Wang, K.; Yan, W. Adsorption and Removal Mechanism of Pb(II) by Oxidized Multi-Walled Carbon Nanotubes. *Journal of the Iranian Chemical Society* **2022**, *19*, doi:10.1007/s13738-022-02502-1.
83. Yang, P.; Li, F.; Wang, B.; Niu, Y.; Wei, J.; Yu, Q. In Situ Synthesis of Carbon Nanotube–Steel Slag Composite for Pb(II) and Cu(II) Removal from Aqueous Solution. *Nanomaterials* **2022**, *12*, doi:10.3390/nano12071199.
84. Salem, M.A.S.; Khan, A.M.; Manea, Y.K.; Wani, A.A. Nano Chromium Embedded in F-CNT Supported CoBi-LDH Nanocomposites for Selective Adsorption of Pb2+and Hazardous Organic Dyes. *Chemosphere* **2022**, *289*, doi:10.1016/j.chemosphere.2021.133073.
85. Zondo, B.Z.; Sadare, O.O.; Simate, G.S.; Moothi, K. Removal of Pb2+ Ions from Synthetic Wastewater Using Functionalized Multi-Walled Carbon Nanotubes Decorated with Green Synthesized Iron Oxide–Gold Nanocomposite. *Water SA* **2022**, *48*, doi:10.17159/wsa/2022.v48.i3.3959.
86. Veličković, Z.S.; Marinković, A.D.; Bajić, Z.J.; Marković, J.M.; Perić-Grujić, A.A.; Uskokovic, P.S.; Ristic, M.D. Oxidized and Ethylenediamine-Functionalized Multi-Walled Carbon Nanotubes for the Separation of Low Concentration Arsenate from Water. *Separation Science and Technology (Philadelphia)* **2013**, *48*, doi:10.1080/01496395.2013.790446.
87. Chen, B.; Zhu, Z.; Ma, J.; Qiu, Y.; Chen, J. Surfactant Assisted Ce-Fe Mixed Oxide Decorated Multiwalled Carbon Nanotubes and Their Arsenic Adsorption Performance. *J Mater Chem A Mater* **2013**, *1*, doi:10.1039/c3ta11827d.
88. Kónya, Z.; Vesselenyi, I.; Niesz, K.; Kukovecz, A.; Demortier, A.; Fonseca, A.; Delhalle, J.; Mekhalif, Z.; Nagy, J.B.; Koós, A.A.; et al. Large Scale Production of Short Functionalized Carbon Nanotubes. *Chem Phys Lett* **2002**, *360*, doi:10.1016/S0009-2614(02)00900-4.
89. Sankararamakrishnan, N.; Gupta, A.; Vidyarthi, S.R. Enhanced Arsenic Removal at Neutral PH Using Functionalized Multiwalled Carbon Nanotubes. *J Environ Chem Eng* **2014**, *2*, doi:10.1016/j.jece.2014.02.010.
90. Ma, M.D.; Wu, H.; Deng, Z.Y.; Zhao, X. Arsenic Removal from Water by Nanometer Iron Oxide Coated Single-Wall Carbon Nanotubes. *J Mol Liq* **2018**, *259*, doi:10.1016/j.molliq.2018.03.052.
91. Agrawal, P.R.; Singh, N.; Kumari, S.; Dhakate, S.R. Multiwall Carbon Nanotube Embedded Phenolic Resin-Based Carbon Foam for the Removal of As (V) from Contaminated Water. *Mater Res Express* **2018**, *5*, doi:10.1088/2053-1591/aaaf7c.
92. Akha, N.Z.; Salehi, S.; Anbia, M. Removal of Arsenic by Metal Organic Framework/Chitosan/Carbon Nanocomposites: Modeling, Optimization, and Adsorption Studies. *Int J Biol Macromol* **2022**, *208*, doi:10.1016/j.ijbiomac.2022.03.161.
93. Wang, Z.; Fang, Y.; Yang, Y.; Qiu, B.; Li, H. Vacancies-Rich MOFs-Derived Magnetic CoFe Encapsulated in N-Doped Carbon Nanotubes as Peroxymonosulfate Activator for p-Arsanilic Acid Removal. *Chemical Engineering Journal* **2023**, *454*, doi:10.1016/j.cej.2022.140474.
94. Ali, I. Microwave Assisted Economic Synthesis of Multi Walled Carbon Nanotubes for Arsenic Species Removal in Water: Batch and Column Operations. *J Mol Liq* **2018**, *271*, doi:10.1016/j.molliq.2018.09.021.
95. Liu, G.; Li, L.; Huang, X.; Zheng, S.; Xu, X.; Liu, Z.; Zhang, Y.; Wang, J.; Lin, H.; Xu, D. Adsorption and Removal of Organophosphorus Pesticides from Environmental Water and Soil Samples by Using Magnetic Multi-Walled Carbon Nanotubes @ Organic Framework ZIF-8. *J Mater Sci* **2018**, *53*, doi:10.1007/s10853-018-2352-y.
96. Alimohammady, M.; Jahangiri, M.; Kiani, F.; Tahermansouri, H. Design and Evaluation of Functionalized Multi-Walled Carbon Nanotubes by 3-Aminopyrazole for the Removal of Hg(II) and As(III) Ions from Aqueous Solution. *Research on Chemical Intermediates* **2018**, *44*, doi:10.1007/s11164-017-3091-4.

97. Neelgund, G.M.; Aguilar, S.F.; Kurkuri, M.D.; Rodrigues, D.F.; Ray, R.L. Elevated Adsorption of Lead and Arsenic over Silver Nanoparticles Deposited on Poly(Amidoamine) Grafted Carbon Nanotubes. *Nanomaterials* **2022**, *12*, doi:10.3390/nano12213852.
98. Ge, Y.; Li, Z.; Xiao, D.; Xiong, P.; Ye, N. Sulfonated Multi-Walled Carbon Nanotubes for the Removal of Copper (II) from Aqueous Solutions. *Journal of Industrial and Engineering Chemistry* **2014**, *20*, doi:10.1016/j.jiec.2013.08.030.
99. Dichiara, A.B.; Webber, M.R.; Gorman, W.R.; Rogers, R.E. Removal of Copper Ions from Aqueous Solutions via Adsorption on Carbon Nanocomposites. *ACS Appl Mater Interfaces* **2015**, *7*, doi:10.1021/acsami.5b04974.
100. Li, Q.; Yu, J.; Zhou, F.; Jiang, X. Synthesis and Characterization of Dithiocarbamate Carbon Nanotubes for the Removal of Heavy Metal Ions from Aqueous Solutions. *Colloids Surf A Physicochem Eng Asp* **2015**, *482*, doi:10.1016/j.colsurfa.2015.06.034.
101. Bankole, M.T.; Abdulkareem, A.S.; Mohammed, I.A.; Ochigbo, S.S.; Tijani, J.O.; Abubakre, O.K.; Roos, W.D. Selected Heavy Metals Removal From Electroplating Wastewater by Purified and Polyhydroxylbutyrate Functionalized Carbon Nanotubes Adsorbents. *Sci Rep* **2019**, *9*, doi:10.1038/s41598-018-37899-4.
102. Yang, W.; Ding, P.; Zhou, L.; Yu, J.; Chen, X.; Jiao, F. Preparation of Diamine Modified Mesoporous Silica on Multi-Walled Carbon Nanotubes for the Adsorption of Heavy Metals in Aqueous Solution. *Appl Surf Sci* **2013**, *282*, doi:10.1016/j.apsusc.2013.05.028.
103. Venkata Ramana, D.K.; Yu, J.S.; Seshaiah, K. Silver Nanoparticles Deposited Multiwalled Carbon Nanotubes for Removal of Cu(II) and Cd(II) from Water: Surface, Kinetic, Equilibrium, and Thermal Adsorption Properties. *Chemical Engineering Journal* **2013**, *223*, doi:10.1016/j.cej.2013.03.001.
104. Popuri, S.R.; Frederick, R.; Chang, C.Y.; Fang, S.S.; Wang, C.C.; Lee, L.C. Removal of Copper (II) Ions from Aqueous Solutions onto Chitosan/Carbon Nanotubes Composite Sorbent. *Desalination Water Treat* **2014**, *52*, doi:10.1080/19443994.2013.826779.
105. Sobhanardakani, S.; Zandipak, R.; Cheraghi, M. Adsorption of Cu2+ Ions From Aqueous Solutions Using Oxidized Multi-Walled Carbon Nanotubes. *Avicenna Journal of Environmental Health Engineering* **2015**, *2*, doi:10.17795/ajehe790.
106. Ma, J.; Zhuang, Y.; Yu, F. Facile Method for the Synthesis of a Magnetic CNTs-C@Fe-Chitosan Composite and Its Application in Tetracycline Removal from Aqueous Solutions. *Physical Chemistry Chemical Physics* **2015**, *17*, doi:10.1039/c5cp02542g.
107. Zhao, X.H.; Jiao, F.P.; Yu, J.G.; Xi, Y.; Jiang, X.Y.; Chen, X.Q. Removal of Cu(II) from Aqueous Solutions by Tartaric Acid Modified Multi-Walled Carbon Nanotubes. *Colloids Surf A Physicochem Eng Asp* **2015**, *476*, doi:10.1016/j.colsurfa.2015.03.016.
108. Tofighy, M.A.; Mohammadi, T. Copper Ions Removal from Aqueous Solutions Using Acid-Chitosan Functionalized Carbon Nanotubes Sheets. *Desalination Water Treat* **2016**, *57*, doi:10.1080/19443994.2015.1072738.
109. Salam, M.A.; Al-Zhrani, G.; Kosa, S.A. Simultaneous Removal of Copper(II), Lead(II), Zinc(II) and Cadmium(II) from Aqueous Solutions by Multi-Walled Carbon Nanotubes. *Comptes Rendus Chimie* **2012**, *15*, doi:10.1016/j.crci.2012.01.013.
110. Abdel Salam, E.T.; Abou El-Nour, K.M.; Awad, A.A.; Orabi, A.S. Carbon Nanotubes Modified with 5,7-Dinitro-8-Quinolinol as Potentially Applicable Tool for Efficient Removal of Industrial Wastewater Pollutants. *Arabian Journal of Chemistry* **2020**, *13*, doi:10.1016/j.arabjc.2017.02.005.
111. Abdulkareem, A.S.; Hamzat, W.A.; Tijani, J.O.; Egbosiuba, T.C.; Mustapha, S.; Abubakre, O.K.; Okafor, B.O.; Babayemi, A.K. Isotherm, Kinetics, Thermodynamics and Mechanism of Metal Ions Adsorption from Electroplating Wastewater Using Treated and Functionalized Carbon Nanotubes. *J Environ Chem Eng* **2023**, *11*, doi:10.1016/j.jece.2022.109180.

112. Abo-Zahra, S.F.; Abdelmonem, I.M.; Siyam, T.E.; El-Masry, A.M.; Abdel-Aziz, H.M. Radiation Synthesis of Polyacrylamide/Functionalized Multiwalled Carbon Nanotubes Composites for the Adsorption of Cu(II) Metal Ions from Aqueous Solution. *Polymer Bulletin* **2022**, *79*, doi:10.1007/s00289-021-03726-6.
113. Ghanavati, B.; Bozorgian, A.; Ghanavati, J. Removal of Copper (II) Ions from the Effluent by Carbon Nanotubes Modified with Tetrahydrofuran. *Chemical Review and Letters* **2022**, *5*, doi:10.22034/CRL.2022.326950.1152.
114. Dou, J.; Gan, D.; Huang, Q.; Liu, M.; Chen, J.; Deng, F.; Zhu, X.; Wen, Y.; Zhang, X.; Wei, Y. Functionalization of Carbon Nanotubes with Chitosan Based on MALI Multicomponent Reaction for Cu2+ Removal. *Int J Biol Macromol* **2019**, *136*, doi:10.1016/j.ijbiomac.2019.06.112.
115. Zhou, Y.; He, Y.; Xiang, Y.; Meng, S.; Liu, X.; Yu, J.; Yang, J.; Zhang, J.; Qin, P.; Luo, L. Single and Simultaneous Adsorption of Pefloxacin and Cu(II) Ions from Aqueous Solutions by Oxidized Multiwalled Carbon Nanotube. *Science of the Total Environment* **2019**, *646*, doi:10.1016/j.scitotenv.2018.07.267.
116. Rodríguez, C.; Briano, S.; Leiva, E. Increased Adsorption of Heavy Metal Ions in Multi-Walled Carbon Nanotubes with Improved Dispersion Stability. *Molecules* **2020**, *25*, doi:10.3390/molecules25143106.
117. Mwafy, E.A.; Mostafa, A.M. Tailored MWCNTs/SnO2 Decorated Cellulose Nanofiber Adsorbent for the Removal of Cu (II) from Waste Water. *Radiation Physics and Chemistry* **2020**, *177*, doi:10.1016/j.radphyschem.2020.109172.
118. Deng, Y.; Ok, Y.S.; Mohan, D.; Pittman, C.U.; Dou, X. Carbamazepine Removal from Water by Carbon Dot-Modified Magnetic Carbon Nanotubes. *Environ Res* **2019**, *169*, doi:10.1016/j.envres.2018.11.035.
119. Serag, E.; El Nemr, A.; Fathy, S.A.; Hamid, F.F.A.; El-Maghraby, A. A Novel Three Dimensional Carbon Nanotube-Polyethylene Glycol-Polyvinyl Alcohol Nanocomposite for Cu(II) Removal from Water. *Egypt J Aquat Biol Fish* **2018**, *22*, doi:10.21608/EJABF.2018.8234.
120. Hosseinzadeh, H.; Pashaei, S.; Hosseinzadeh, S.; Khodaparast, Z.; Ramin, S.; Saadat, Y. Preparation of Novel Multi-Walled Carbon Nanotubes Nanocomposite Adsorbent via RAFT Technique for the Adsorption of Toxic Copper Ions. *Science of the Total Environment* **2018**, *640–641*, doi:10.1016/j.scitotenv.2018.05.326.
121. Liang, J.; Liu, J.; Yuan, X.; Dong, H.; Zeng, G.; Wu, H.; Wang, H.; Liu, J.; Hua, S.; Zhang, S.; et al. Facile Synthesis of Alumina-Decorated Multi-Walled Carbon Nanotubes for Simultaneous Adsorption of Cadmium Ion and Trichloroethylene. *Chemical Engineering Journal* **2015**, *273*, doi:10.1016/j.cej.2015.03.069.
122. Ihsanullah; Al-Khaldi, F.A.; Abusharkh, B.; Khaled, M.; Atieh, M.A.; Nasser, M.S.; Laoui, T.; Saleh, T.A.; Agarwal, S.; Tyagi, I.; et al. Adsorptive Removal of Cadmium(II) Ions from Liquid Phase Using Acid Modified Carbon-Based Adsorbents. *J Mol Liq* **2015**, *204*, doi:10.1016/j.molliq.2015.01.033.
123. Al-Khaldi, F.A.; Abu-Sharkh, B.; Abulkibash, A.M.; Atieh, M.A. Cadmium Removal by Activated Carbon, Carbon Nanotubes, Carbon Nanofibers, and Carbon Fly Ash: A Comparative Study. *Desalination Water Treat* **2015**, *53*, doi:10.1080/19443994.2013.847805.
124. A Akl, M.; Elanwar, A.M.A. Adsorption Studies of Cd (II) from Water by Acid Modified Multiwalled Carbon Nanotubes. *J Nanomed Nanotechnol* **2015**, *06*, doi:10.4172/2157-7439.1000327.
125. Sun, W.; Jiang, B.; Wang, F.; Xu, N. Effect of Carbon Nanotubes on Cd(II) Adsorption by Sediments. *Chemical Engineering Journal* **2015**, *264*, doi:10.1016/j.cej.2014.11.137.
126. Al-Kadhi, N.S.; Pashameah, R.A.; Ahmed, H.A.; Alrefaee, S.H.; Alamro, F.S.; Faqih, H.H.; Mwafy, E.A.; Mostafa, A.M. Preparation of NiO/MWCNTs Nanocomposite for the Removal of Cadmium Ions. *Journal of Materials Research and Technology* **2022**, *19*, doi:10.1016/j.jmrt.2022.05.149.
127. Zhou, W.; Wu, P.; Zhang, L.; Zhu, D.; Zhao, X.; Cai, Y. Heavy Metal Ions and Particulate Pollutants Can Be Effectively Removed by a Gravity-Driven Ceramic Foam Filter Optimized by Carbon Nanotube Implantation. *J Hazard Mater* **2022**, *421*, doi:10.1016/j.jhazmat.2021.126721.

128. Ye, L.; Wang, L.; Wei, Z.; Zhou, S.; Yao, Z.; Fan, F.; Mei, Y. Thin Film Composite Nanofiltration Membrane with Tannic Acid-Fe(III) Complexes Functionalized CNTs Interlayer toward Energy Efficient Remediation of Groundwater. *Desalination* **2023**, *552*, doi:10.1016/j.desal.2023.116438.
129. Adelabu, I.O.; Saleh, T.A.; Garrison, T.F.; Al Hamouz, O.C.S. Synthesis of Polyamine-CNT Composites for the Removal of Toxic Cadmium Metal Ions from Wastewater. *J Mol Liq* **2020**, *297*, doi:10.1016/j.molliq.2019.111827.
130. Jung, C.; Heo, J.; Han, J.; Her, N.; Lee, S.J.; Oh, J.; Ryu, J.; Yoon, Y. Hexavalent Chromium Removal by Various Adsorbents: Powdered Activated Carbon, Chitosan, and Single/Multi-Walled Carbon Nanotubes. *Sep Purif Technol* **2013**, *106*, doi:10.1016/j.seppur.2012.12.028.
131. Sankararamakrishnan, N.; Jaiswal, M.; Verma, N. Composite Nanofloral Clusters of Carbon Nanotubes and Activated Alumina: An Efficient Sorbent for Heavy Metal Removal. *Chemical Engineering Journal* **2014**, *235*, doi:10.1016/j.cej.2013.08.070.
132. Krishna Kumar, A.S.; Jiang, S.J.; Tseng, W.L. Effective Adsorption of Chromium(vi)/Cr(Iii) from Aqueous Solution Using Ionic Liquid Functionalized Multiwalled Carbon Nanotubes as a Super Sorbent. *J Mater Chem A Mater* **2015**, *3*, doi:10.1039/c4ta06948j.
133. Ihsanullah; Abbas, A.; Al-Amer, A.M.; Laoui, T.; Al-Marri, M.J.; Nasser, M.S.; Khraisheh, M.; Atieh, M.A. Heavy Metal Removal from Aqueous Solution by Advanced Carbon Nanotubes: Critical Review of Adsorption Applications. *Sep Purif Technol* 2016, *157*.
134. Ansari, M.O.; Kumar, R.; Ansari, S.A.; Ansari, S.P.; Barakat, M.A.; Alshahrie, A.; Cho, M.H. Anion Selective PTSA Doped Polyaniline@graphene Oxide-Multiwalled Carbon Nanotube Composite for Cr(VI) and Congo Red Adsorption. *J Colloid Interface Sci* **2017**, *496*, doi:10.1016/j.jcis.2017.02.034.
135. Huang, Z. nan; Wang, X. ling; Yang, D. suo Adsorption of Cr(VI) in Wastewater Using Magnetic Multi-Wall Carbon Nanotubes. *Water Science and Engineering* **2015**, *8*, doi:10.1016/j.wse.2015.01.009.
136. Bayazit, Ş.S.; Kerkez, Ö. Hexavalent Chromium Adsorption on Superparamagnetic Multi-Wall Carbon Nanotubes and Activated Carbon Composites. *Chemical Engineering Research and Design* **2014**, *92*, doi:10.1016/j.cherd.2014.02.007.
137. Hossini, H.; Rezaee, A.; Rastegar, S.O.; Hashemi, S.; Safari, M. Equilibrium and Kinetic Studies of Chromium Adsorption from Wastewater by Functionalized Multi-Wall Carbon Nanotubes. *Reaction Kinetics, Mechanisms and Catalysis* **2014**, *112*, doi:10.1007/s11144-014-0699-x.
138. Hao, P.; Ma, X.; Xie, J.; Lei, F.; Li, L.; Zhu, W.; Cheng, X.; Cui, G.; Tang, B. Removal of Toxic Metal Ions Using Chitosan Coated Carbon Nanotube Composites for Supercapacitors. *Sci China Chem* **2018**, *61*, doi:10.1007/s11426-017-8215-7.
139. Liu, K.; Zhao, D.; Hu, Z.; Xiao, Y.; He, C.; Jiang, F.; Zhao, N.; Zhao, C.; Zhang, W.; Qiu, R. The Adsorption and Reduction of Anionic Cr(VI) in Groundwater by Novel Iron Carbide Loaded on N-Doped Carbon Nanotubes: Effects of Fe-Confinement. *Chemical Engineering Journal* **2023**, *452*, doi:10.1016/j.cej.2022.139357.
140. Chen, Z.; Fu, D.; Yuen Koh, K.; Paul Chen, J. A New Carbon Nanotube Modified by Nano CaO2 for Removal of Chromate and Phosphate from Aqueous Solutions. *Chemical Engineering Journal* **2022**, *446*, doi:10.1016/j.cej.2022.136845.
141. Amaku, J.F.; Ogundare, S.A.; Akpomie, K.G.; Ngwu, C.M.; Conradie, J. Enhanced Chromium (VI) Removal by Anacardium Occidentale Stem Bark Extract-Coated Multiwalled Carbon Nanotubes. *International Journal of Environmental Science and Technology* **2022**, *19*, doi:10.1007/s13762-021-03364-5.
142. Wang, Y.; Bailey, J.; Zhu, Y.; Zhang, Y.; Boetcher, S.K.S.; Li, Y.; Wu, C. Application of Carbon Nanotube Prepared from Waste Plastic to Phase Change Materials: The Potential for Battery Thermal Management. *Waste Management* **2022**, *154*, doi:10.1016/j.wasman.2022.10.003.

143. Huang, Y.; Lee, X.; Macazo, F.C.; Grattieri, M.; Cai, R.; Minteer, S.D. Fast and Efficient Removal of Chromium (VI) Anionic Species by a Reusable Chitosan-Modified Multi-Walled Carbon Nanotube Composite. *Chemical Engineering Journal* **2018**, *339*, doi:10.1016/j.cej.2018.01.133.
144. Yao, Y.; Zhang, J.; Chen, H.; Yu, M.; Gao, M.; Hu, Y.; Wang, S. Ni 0 Encapsulated in N-Doped Carbon Nanotubes for Catalytic Reduction of Highly Toxic Hexavalent Chromium. *Appl Surf Sci* **2018**, *440*, doi:10.1016/j.apsusc.2018.01.123.
145. Mubarak, N.M.; Alicia, R.F.; Abdullah, E.C.; Sahu, J.N.; Haslija, A.B.A.; Tan, J. Statistical Optimization and Kinetic Studies on Removal of Zn2+ Using Functionalized Carbon Nanotubes and Magnetic Biochar. *J Environ Chem Eng* **2013**, *1*, doi:10.1016/j.jece.2013.06.011.
146. Park, W.K.; Yoon, Y.; Kim, S.; Yoo, S.; Do, Y.; Kang, J.W.; Yoon, D.H.; Yang, W.S. Feasible Water Flow Filter with Facilely Functionalized Fe3O4-Non-Oxidative Graphene/CNT Composites for Arsenic Removal. *J Environ Chem Eng* **2016**, *4*, doi:10.1016/j.jece.2016.06.028.
147. Ali, S.; Shah, I.A.; Ahmad, A.; Nawab, J.; Huang, H. Ar/O2 Plasma Treatment of Carbon Nanotube Membranes for Enhanced Removal of Zinc from Water and Wastewater: A Dynamic Sorption-Filtration Process. *Science of the Total Environment* **2019**, *655*, doi:10.1016/j.scitotenv.2018.11.335.
148. Hayati, B.; Maleki, A.; Najafi, F.; Daraei, H.; Gharibi, F.; McKay, G. Synthesis and Characterization of PAMAM/CNT Nanocomposite as a Super-Capacity Adsorbent for Heavy Metal (Ni2 +, Zn2 +, As3 +, Co2 +) Removal from Wastewater. *J Mol Liq* **2016**, *224*, doi:10.1016/j.molliq.2016.10.053.
149. Abdel-Ghani, N.T.; El-Chaghaby, G.A.; Helal, F.S. Individual and Competitive Adsorption of Phenol and Nickel onto Multiwalled Carbon Nanotubes. *J Adv Res* **2015**, *6*, doi:10.1016/j.jare.2014.06.001.
150. Egbosiuba, T.C.; Abdulkareem, A.S.; Tijani, J.O.; Ani, J.I.; Krikstolaityte, V.; Srinivasan, M.; Veksha, A.; Lisak, G. Taguchi Optimization Design of Diameter-Controlled Synthesis of Multi Walled Carbon Nanotubes for the Adsorption of Pb(II) and Ni(II) from Chemical Industry Wastewater. *Chemosphere* **2021**, *266*, doi:10.1016/j.chemosphere.2020.128937.
151. Liu, Z.; Chen, L.; Zhang, Z.; Li, Y.; Dong, Y.; Sun, Y. Synthesis of Multi-Walled Carbon Nanotube-Hydroxyapatite Composites and Its Application in the Sorption of Co(II) from Aqueous Solutions. *J Mol Liq* **2013**, *179*, doi:10.1016/j.molliq.2012.12.011.
152. Karkeh-abadi, F.; Saber-Samandari, S.; Saber-Samandari, S. The Impact of Functionalized CNT in the Network of Sodium Alginate-Based Nanocomposite Beads on the Removal of Co(II) Ions from Aqueous Solutions. *J Hazard Mater* **2016**, *312*, doi:10.1016/j.jhazmat.2016.03.074.
153. Yang, J.; Dong, Y.; Li, J.; Liu, Z.; Min, F.; Li, Y. Removal of Co(II) from Aqueous Solutions by Sulfonated Magnetic Multi-Walled Carbon Nanotubes. *Korean Journal of Chemical Engineering* **2015**, *32*, doi:10.1007/s11814-015-0072-4.
154. Dehghani, M.H.; Yetilmezsoy, K.; Salari, M.; Heidarinejad, Z.; Yousefi, M.; Sillanpää, M. Adsorptive Removal of Cobalt(II) from Aqueous Solutions Using Multi-Walled Carbon Nanotubes and γ-Alumina as Novel Adsorbents: Modelling and Optimization Based on Response Surface Methodology and Artificial Neural Network. *J Mol Liq* **2020**, *299*, doi:10.1016/j.molliq.2019.112154.
155. Mubarak, N.M.; Sahu, J.N.; Abdullah, E.C.; Jayakumar, N.S. Rapid Adsorption of Toxic Pb(II) Ions from Aqueous Solution Using Multiwall Carbon Nanotubes Synthesized by Microwave Chemical Vapor Deposition Technique. *J Environ Sci (China)* **2016**, *45*, doi:10.1016/j.jes.2015.12.025.
156. Mubarak, N.M.; Sahu, J.N.; Abdullah, E.C.; Jayakumar, N.S.; Ganesan, P. Novel Microwave-Assisted Multiwall Carbon Nanotubes Enhancing Cu (II) Adsorption Capacity in Water. *J Taiwan Inst Chem Eng* **2015**, *53*, doi:10.1016/j.jtice.2015.02.016.
157. Gupta, V.K.; Agarwal, S.; Bharti, A.K.; Sadegh, H. Adsorption Mechanism of Functionalized Multi-Walled Carbon Nanotubes for Advanced Cu (II) Removal. *J Mol Liq* **2017**, *230*, doi:10.1016/j.molliq.2017.01.083.

158. Abu Bakar, S.; Jusoh, N.; Mohamed, A.; Muqoyyanah, M.; Othman, M.H.D.; Mamat, M.H.; Ahmad, M.K.; Mohamed, M.A.; Azlan, M.N.; Hashim, N.; et al. Carbon Nanotubes from Waste Cooking Palm Oil as Adsorbent Materials for the Adsorption of Heavy Metal Ions. *Environmental Science and Pollution Research* **2021**, *28*, doi:10.1007/s11356-021-14918-y.
159. Gusain, R.; Kumar, N.; Fosso-Kankeu, E.; Ray, S.S. Efficient Removal of Pb(II) and Cd(II) from Industrial Mine Water by a Hierarchical MoS2/SH-MWCNT Nanocomposite. *ACS Omega* **2019**, *4*, doi:10.1021/acsomega.9b01603.
160. Zhang, Y.; Yang, J.; Zhong, L.; Liu, L. Effect of Multi-Wall Carbon Nanotubes on Cr(VI) Reduction by Citric Acid: Implications for Their Use in Soil Remediation. *Environmental Science and Pollution Research* **2018**, *25*, doi:10.1007/s11356-018-2438-8.
161. Barrejón, M.; Syrgiannis, Z.; Burian, M.; Bosi, S.; Montini, T.; Fornasiero, P.; Amenitsch, H.; Prato, M. Cross-Linked Carbon Nanotube Adsorbents for Water Treatment: Tuning the Sorption Capacity through Chemical Functionalization. *ACS Appl Mater Interfaces* **2019**, *11*, doi:10.1021/acsami.8b20557.
162. Ncibi, M.C.; Sillanpää, M. Optimized Removal of Antibiotic Drugs from Aqueous Solutions Using Single, Double and Multi-Walled Carbon Nanotubes. *J Hazard Mater* **2015**, *298*, doi:10.1016/j.jhazmat.2015.05.025.
163. Ferreira, G.M.D.; Ferreira, G.M.D.; Hespanhol, M.C.; de Paula Rezende, J.; dos Santos Pires, A.C.; Gurgel, L.V.A.; da Silva, L.H.M. Adsorption of Red Azo Dyes on Multi-Walled Carbon Nanotubes and Activated Carbon: A Thermodynamic Study. *Colloids Surf A Physicochem Eng Asp* **2017**, *529*, doi:10.1016/j.colsurfa.2017.06.021.
164. Álvarez-Torrellas, S.; Rodríguez, A.; Ovejero, G.; García, J. Comparative Adsorption Performance of Ibuprofen and Tetracycline from Aqueous Solution by Carbonaceous Materials. *Chemical Engineering Journal* **2016**, *283*, doi:10.1016/j.cej.2015.08.023.
165. Hanbali, G.; Jodeh, S.; Hamed, O.; Bol, R.; Khalaf, B.; Qdemat, A.; Samhan, S. Enhanced Ibuprofen Adsorption and Desorption on Synthesized Functionalized Magnetic Multiwall Carbon Nanotubes from Aqueous Solution. *Materials* **2020**, *13*, doi:10.3390/ma13153329.
166. Ma, H.; Zhang, X.; Feng, G.; Ren, B.; Pan, Z.; Shi, Y.; Xu, R.; Wang, P.; Liu, Y.; Wang, G.; et al. Carbon Nanotube Membrane Armed with Confined Iron for Peroxymonosulfate Activation towards Efficient Tetracycline Removal. *Sep Purif Technol* **2023**, *312*, doi:10.1016/j.seppur.2023.123319.
167. Yang, Q.; Chen, G.; Zhang, J.; Li, H. Adsorption of Sulfamethazine by Multi-Walled Carbon Nanotubes: Effects of Aqueous Solution Chemistry. *RSC Adv* **2015**, *5*, doi:10.1039/c4ra15056b.
168. Moradi, O. Adsorption Behavior of Basic Red 46 by Single-Walled Carbon Nanotubes Surfaces. *Fullerenes Nanotubes and Carbon Nanostructures* **2013**, *21*, doi:10.1080/1536383X.2011.572317.
169. Shan, D.; Deng, S.; Zhao, T.; Yu, G.; Winglee, J.; Wiesner, M.R. Preparation of Regenerable Granular Carbon Nanotubes by a Simple Heating-Filtration Method for Efficient Removal of Typical Pharmaceuticals. *Chemical Engineering Journal* **2016**, *294*, doi:10.1016/j.cej.2016.02.118.
170. Czech, B. The Effect of MWCNT Treatment by H2O2 and/or UV on Fulvic Acids Sorption. *Environ Res* **2017**, *155*, doi:10.1016/j.envres.2017.01.037.
171. Ateia, M.; Ceccato, M.; Budi, A.; Ataman, E.; Yoshimura, C.; Johnson, M.S. Ozone-Assisted Regeneration of Magnetic Carbon Nanotubes for Removing Organic Water Pollutants. *Chemical Engineering Journal* **2018**, *335*, doi:10.1016/j.cej.2017.10.166.
172. Madihi-Bidgoli, S.; Asadnezhad, S.; Yaghoot-Nezhad, A.; Hassani, A. Azurobine Degradation Using Fe2O3@multi-Walled Carbon Nanotube Activated Peroxymonosulfate (PMS) under UVA-LED Irradiation: Performance, Mechanism and Environmental Application. *J Environ Chem Eng* **2021**, *9*, doi:10.1016/j.jece.2021.106660.
173. Duman, O.; Özcan, C.; Gürkan Polat, T.; Tunç, S. Carbon Nanotube-Based Magnetic and Non-Magnetic Adsorbents for the High-Efficiency Removal of Diquat Dibromide Herbicide from Water: OMWCNT, OMWCNT-

Fe3O4 and OMWCNT-K-Carrageenan-Fe3O4 Nanocomposites. *Environmental Pollution* **2019**, *244*, doi:10.1016/j.envpol.2018.10.071.

174. Liu, W.; Li, Z.; Zhang, S.P.; Jian, W.W.; Ma, D.Z. Adsorption Performance of Multi-Walled Carbon Nanotube-SiO2 Adsorbent for Toluene. *Ranliao Huaxue Xuebao/Journal of Fuel Chemistry and Technology* **2021**, *49*, doi:10.1016/S1872-5813(21)60090-7.

175. Lung, I.; Soran, M.L.; Stegarescu, A.; Opriş, O. Application of CNT-COOH/MnO2/Fe3O4 Nanocomposite for the Removal of Cymoxanil from Aqueous Solution: Isotherm and Kinetic Studies. *Anal Lett* **2023**, *56*, doi:10.1080/00032719.2022.2043888.

176. Zahedinejad, M.; Sohrabi, N.; Mohammadi, R. Magnetic Multi-Walled Carbon Nanotubes as an Efficient Sorbent for Pirimicarb Removal from Aqueous Solutions in Continuous (FBAC) and Batch Formats: Thermodynamic, Kinetic, Isotherm Study, Optimization and Modeling by RSM-ANN. *J Mol Liq* **2023**, *370*, doi:10.1016/j.molliq.2022.120915.

177. Massad, Y.; Hanbali, G.; Jodeh, S.; Hamed, O.; Bzour, M.; Dagdag, O.; Samhan, S. The Efficiency of Removal of Organophosphorus Malathion Pesticide Using Functionalized Multi-Walled Carbon Nanotube: Impact of Dissolved Organic Matter (DOM). *Separation Science and Technology (Philadelphia)* **2022**, *57*, doi:10.1080/01496395.2021.1881118.

178. Zare, K.; Sadegh, H.; Shahryari-Ghoshekandi, R.; Maazinejad, B.; Ali, V.; Tyagi, I.; Agarwal, S.; Gupta, V.K. Enhanced Removal of Toxic Congo Red Dye Using Multi Walled Carbon Nanotubes: Kinetic, Equilibrium Studies and Its Comparison with Other Adsorbents. *J Mol Liq* **2015**, *212*, doi:10.1016/j.molliq.2015.09.027.

179. Vuono, D.; Catizzone, E.; Aloise, A.; Policicchio, A.; Agostino, R.G.; Migliori, M.; Giordano, G. Study of Adsorption Behavior of Multi-Walled Carbon Nanotubes towards Dyes Applied in Textile Applications. *Adv Sci Lett* **2017**, *23*, doi:10.1166/asl.2017.9048.

180. Robati, D.; Bagheriyan, S.; Rajabi, M.; Moradi, O.; Peyghan, A.A. Effect of Electrostatic Interaction on the Methylene Blue and Methyl Orange Adsorption by the Pristine and Functionalized Carbon Nanotubes. *Physica E Low Dimens Syst Nanostruct* **2016**, *83*, doi:10.1016/j.physe.2016.04.005.

181. Abbasi, M. Synthesis and Characterization of Magnetic Nanocomposite of Chitosan/SiO2/Carbon Nanotubes and Its Application for Dyes Removal. *J Clean Prod* **2017**, *145*, doi:10.1016/j.jclepro.2017.01.046.

182. Mohammadi, A.; Veisi, P. High Adsorption Performance of β-Cyclodextrin-Functionalized Multi-Walled Carbon Nanotubes for the Removal of Organic Dyes from Water and Industrial Wastewater. *J Environ Chem Eng* **2018**, *6*, doi:10.1016/j.jece.2018.07.002.

183. Hosseinzadeh, S.; Hosseinzadeh, H.; Pashaei, S.; Khodaparast, Z. Synthesis of Magnetic Functionalized MWCNT Nanocomposite through Surface RAFT Co-Polymerization of Acrylic Acid and N-Isopropyl Acrylamide for Removal of Cationic Dyes from Aqueous Solutions. *Ecotoxicol Environ Saf* **2018**, *161*, doi:10.1016/j.ecoenv.2018.05.063.

184. Saber-Samandari, S.; Saber-Samandari, S.; Joneidi-Yekta, H.; Mohseni, M. Adsorption of Anionic and Cationic Dyes from Aqueous Solution Using Gelatin-Based Magnetic Nanocomposite Beads Comprising Carboxylic Acid Functionalized Carbon Nanotube. *Chemical Engineering Journal* **2017**, *308*, doi:10.1016/j.cej.2016.10.017.

185. Wongaree, M.; Chiarakorn, S.; Chuangchote, S. Photocatalytic Improvement under Visible Light in TiO2 Nanoparticles by Carbon Nanotube Incorporation. *J Nanomater* **2015**, *2015*, doi:10.1155/2015/689306.

186. Xu, Y.; Xu, H.; Wang, L.; Yan, J.; Li, H.; Song, Y.; Huang, L.; Cai, G. The CNT Modified White C3N4 Composite Photocatalyst with Enhanced Visible-Light Response Photoactivity. *Journal of the Chemical Society. Dalton Transactions* **2013**, *42*, doi:10.1039/c3dt32871f.

187. Wang, S.; Zhou, S. Photodegradation of Methyl Orange by Photocatalyst of CNTs/P-TiO2 under UV and Visible-Light Irradiation. *J Hazard Mater* **2011**, *185*, doi:10.1016/j.jhazmat.2010.08.125.

188. Samsudin, M.F.R.; Bacho, N.; Sufian, S.; Ng, Y.H. Photocatalytic Degradation of Phenol Wastewater over Z-Scheme g-C 3 N 4 /CNT/BiVO 4 Heterostructure Photocatalyst under Solar Light Irradiation. *J Mol Liq* **2019**, *277*, doi:10.1016/j.molliq.2018.10.160.
189. Nik Yusoff, N.R.; Lim, P.W.; Azmi, N.A.B.; Yusoff, M.; Muhammad, M. Effect of Photocatalyst Dosage and Air Loading in Photocatalytic Degradation of Metamifop. In Proceedings of the IOP Conference Series: Earth and Environmental Science; 2021; Vol. 842.
190. Mohamed, A.; Yousef, S.; Nasser, W.S.; Osman, T.A.; Knebel, A.; Sánchez, E.P.V.; Hashem, T. Rapid Photocatalytic Degradation of Phenol from Water Using Composite Nanofibers under UV. *Environ Sci Eur* **2020**, *32*, doi:10.1186/s12302-020-00436-0.
191. Mahmoodi, N.M.; Rezaei, P.; Ghotbei, C.; Kazemeini, M. Copper Oxide-Carbon Nanotube (CuO/CNT) Nanocomposite: Synthesis and Photocatalytic Dye Degradation from Colored Textile Wastewater. *Fibers and Polymers* **2016**, *17*, doi:10.1007/s12221-016-6645-y.
192. Akter, J.; Hanif, M.A.; Islam, M.A.; Sapkota, K.P.; Hahn, J.R. Selective Growth of Ti3+/TiO2/CNT and Ti3+/TiO2/C Nanocomposite for Enhanced Visible-Light Utilization to Degrade Organic Pollutants by Lowering TiO2-Bandgap. *Sci Rep* **2021**, *11*, doi:10.1038/s41598-021-89026-5.
193. Czech, B.; Buda, W. Photocatalytic Treatment of Pharmaceutical Wastewater Using New Multiwall-Carbon Nanotubes/TiO2/SiO2 Nanocomposites. *Environ Res* **2015**, *137*, doi:10.1016/j.envres.2014.12.006.
194. Yibeltal, A.W.; Beyene, B.B.; Admassie, S.; Taddesse, A.M. MWCNTs/Ag-ZnO Nanocomposite for Efficient Photocatalytic Degradation of Congo Red. *Bull Chem Soc Ethiop* **2020**, *34*, doi:10.4314/BCSE.V34I1.5.
195. Li, S.; Liao, G.; Liu, Z.; Pan, Y.; Wu, Q.; Weng, Y.; Zhang, X.; Yang, Z.; Tsui, O.K.C. Enhanced Water Flux in Vertically Aligned Carbon Nanotube Arrays and Polyethersulfone Composite Membranes. *J Mater Chem A Mater* **2014**, *2*, doi:10.1039/c4ta02119c.
196. Baek, Y.; Kim, C.; Seo, D.K.; Kim, T.; Lee, J.S.; Kim, Y.H.; Ahn, K.H.; Bae, S.S.; Lee, S.C.; Lim, J.; et al. High Performance and Antifouling Vertically Aligned Carbon Nanotube Membrane for Water Purification. *J Memb Sci* **2014**, *460*, doi:10.1016/j.memsci.2014.02.042.
197. Lee, C.; Baik, S. Vertically-Aligned Carbon Nano-Tube Membrane Filters with Superhydrophobicity and Superoleophilicity. *Carbon N Y* **2010**, *48*, doi:10.1016/j.carbon.2010.02.020.
198. Brady-Estévez, A.S.; Kang, S.; Elimelech, M. A Single-Walled-Carbon-Nanotube Filter for Removal of Viral and Bacterial Pathogens. *Small* **2008**, *4*, doi:10.1002/smll.200700863.
199. Lee, B.; Baek, Y.; Lee, M.; Jeong, D.H.; Lee, H.H.; Yoon, J.; Kim, Y.H. A Carbon Nanotube Wall Membrane for Water Treatment. *Nat Commun* **2015**, *6*, doi:10.1038/ncomms8109.
200. Park, S.M.; Jung, J.; Lee, S.; Baek, Y.; Yoon, J.; Seo, D.K.; Kim, Y.H. Fouling and Rejection Behavior of Carbon Nanotube Membranes. *Desalination* **2014**, *343*, doi:10.1016/j.desal.2013.10.005.
201. Matsumoto, H.; Tsuruoka, S.; Hayashi, Y.; Abe, K.; Hata, K.; Zhang, S.; Saito, Y.; Aiba, M.; Tokunaga, T.; Iijima, T.; et al. Water Transport Phenomena through Membranes Consisting of Vertically-Aligned Double-Walled Carbon Nanotube Array. *Carbon N Y* **2017**, *120*, doi:10.1016/j.carbon.2017.05.034.
202. Dumée, L.; Germain, V.; Sears, K.; Schütz, J.; Finn, N.; Duke, M.; Cerneaux, S.; Cornu, D.; Gray, S. Enhanced Durability and Hydrophobicity of Carbon Nanotube Bucky Paper Membranes in Membrane Distillation. *J Memb Sci* **2011**, *376*, doi:10.1016/j.memsci.2011.04.024.
203. Trivedi, S.; Alameh, K. Effect of Vertically Aligned Carbon Nanotube Density on the Water Flux and Salt Rejection in Desalination Membranes. *Springerplus* **2016**, *5*, doi:10.1186/s40064-016-2783-3.
204. Baek, Y.; Seo, D.K.; Choi, J.H.; Lee, B.; Kim, Y.H.; Park, S.M.; Jung, J.; Lee, S.; Yoon, J. Improvement of Vertically Aligned Carbon Nanotube Membranes: Desalination Potential, Flux Enhancement and Scale-Up. *Desalination Water Treat* **2016**, *57*, doi:10.1080/19443994.2016.1184188.

205. Li, K.; Lee, B.; Kim, Y. High Performance Reverse Osmosis Membrane with Carbon Nanotube Support Layer. *J Memb Sci* **2019**, *592*, doi:10.1016/j.memsci.2019.117358.
206. Choi, H.G.; Yoon, S.H.; Son, M.; Celik, E.; Park, H.; Choi, H. Efficacy of Synthesis Conditions on Functionalized Carbon Nanotube Blended Cellulose Acetate Membrane for Desalination. *Desalination Water Treat* **2016**, *57*, doi:10.1080/19443994.2015.1025582.
207. Wang, L.; Song, X.; Wang, T.; Wang, S.; Wang, Z.; Gao, C. Fabrication and Characterization of Polyethersulfone/Carbon Nanotubes (PES/CNTs) Based Mixed Matrix Membranes (MMMs) for Nanofiltration Application. *Appl Surf Sci* **2015**, *330*, doi:10.1016/j.apsusc.2014.12.183.
208. Al-Hobaib, A.S.; Al-Sheetan, K.M.; Shaik, M.R.; Al-Suhybani, M.S. Modification of Thin-Film Polyamide Membrane with Multi-Walled Carbon Nanotubes by Interfacial Polymerization. *Appl Water Sci* **2017**, *7*, doi:10.1007/s13201-017-0578-5.
209. Wan Azelee, I.; Goh, P.S.; Lau, W.J.; Ismail, A.F.; Rezaei-DashtArzhandi, M.; Wong, K.C.; Subramaniam, M.N. Enhanced Desalination of Polyamide Thin Film Nanocomposite Incorporated with Acid Treated Multiwalled Carbon Nanotube-Titania Nanotube Hybrid. *Desalination* **2017**, *409*, doi:10.1016/j.desal.2017.01.029.
210. Gumbi, N.N.; Li, J.; Mamba, B.B.; Nxumalo, E.N. Relating the Performance of Sulfonated Thin-Film Composite Nanofiltration Membranes to Structural Properties of Macrovoid-Free Polyethersulfone/Sulfonated Polysulfone/O-MWCNT Supports. *Desalination* **2020**, *474*, doi:10.1016/j.desal.2019.114176.
211. Ma, X.H.; Guo, H.; Yang, Z.; Yao, Z.K.; Qing, W.H.; Chen, Y.L.; Xu, Z.L.; Tang, C.Y. Carbon Nanotubes Enhance Permeability of Ultrathin Polyamide Rejection Layers. *J Memb Sci* **2019**, *570–571*, doi:10.1016/j.memsci.2018.10.055.
212. Gao, S.; Zhu, Y.; Gong, Y.; Wang, Z.; Fang, W.; Jin, J. Ultrathin Polyamide Nanofiltration Membrane Fabricated on Brush-Painted Single-Walled Carbon Nanotube Network Support for Ion Sieving. *ACS Nano* **2019**, *13*, doi:10.1021/acsnano.8b09761.
213. Fan, X.; Liu, Y.; Quan, X. A Novel Reduced Graphene Oxide/Carbon Nanotube Hollow Fiber Membrane with High Forward Osmosis Performance. *Desalination* **2019**, doi:10.1016/j.desal.2018.07.020.
214. Mahdavi, M.R.; Delnavaz, M.; Vatanpour, V.; Farahbakhsh, J. Effect of Blending Polypyrrole Coated Multiwalled Carbon Nanotube on Desalination Performance and Antifouling Property of Thin Film Nanocomposite Nanofiltration Membranes. *Sep Purif Technol* **2017**, *184*, doi:10.1016/j.seppur.2017.04.037.
215. Vuković, G.D.; Marinković, A.D.; Čolić, M.; Ristić, M.D.; Aleksić, R.; Perić-Grujić, A.A.; Uskoković, P.S. Removal of Cadmium from Aqueous Solutions by Oxidized and Ethylenediamine-Functionalized Multi-Walled Carbon Nanotubes. *Chemical Engineering Journal* **2010**, *157*, doi:10.1016/j.cej.2009.11.026.
216. Li, S.; Gong, Y.; Yang, Y.; He, C.; Hu, L.; Zhu, L.; Sun, L.; Shu, D. Recyclable CNTs/Fe3O4 Magnetic Nanocomposites as Adsorbents to Remove Bisphenol A from Water and Their Regeneration. *Chemical Engineering Journal* **2015**, *260*, doi:10.1016/j.cej.2014.09.032.
217. Wang, W.; Du, Y.; Liu, M.; Yang, M.; Han, R. Adsorption of Congo Red from Solution Using Chitosan Modified Carbon Nanotubes. *Desalination Water Treat* **2019**, *156*, doi:10.5004/dwt.2019.24125.
218. Anjum, H.; Johari, K.; Gnanasundaram, N.; Appusamy, A.; Thanabalan, M. Investigation of Green Functionalization of Multiwall Carbon Nanotubes and Its Application in Adsorption of Benzene, Toluene & p-Xylene from Aqueous Solution. *J Clean Prod* **2019**, *221*, doi:10.1016/j.jclepro.2019.02.233.
219. Xiao, D.; Dramou, P.; Xiong, N.; He, H.; Li, H.; Yuan, D.; Dai, H. Development of Novel Molecularly Imprinted Magnetic Solid-Phase Extraction Materials Based on Magnetic Carbon Nanotubes and Their Application for the Determination of Gatifloxacin in Serum Samples Coupled with High Performance Liquid Chromatography. *J Chromatogr A* **2013**, *1274*, doi:10.1016/j.chroma.2012.12.011.

220. Hsu, S.C.; Lu, C.; Su, F.; Zeng, W.; Chen, W. Thermodynamics and Regeneration Studies of CO2 Adsorption on Multiwalled Carbon Nanotubes. *Chem Eng Sci* **2010**, *65*, doi:10.1016/j.ces.2009.10.005.
221. Toński, M.; Paszkiewicz, M.; Dołżonek, J.; Flejszar, M.; Bielicka-Giełdoń, A.; Stepnowski, P.; Białk-Bielińska, A. Regeneration and Reuse of the Carbon Nanotubes for the Adsorption of Selected Anticancer Drugs from Water Matrices. *Colloids Surf A Physicochem Eng Asp* **2021**, *618*, doi:10.1016/j.colsurfa.2021.126355.
222. Chen, Y.C.; Lu, C. Kinetics, Thermodynamics and Regeneration of Molybdenum Adsorption in Aqueous Solutions with NaOCl-Oxidized Multiwalled Carbon Nanotubes. *Journal of Industrial and Engineering Chemistry* **2014**, *20*, doi:10.1016/j.jiec.2013.10.035.
223. Wang, Y.; Wei, X.; Zhang, R.; Wu, Y.; Farid, M.U.; Huang, H. Comparison of Chemical, Ultrasonic and Thermal Regeneration of Carbon Nanotubes for Acetaminophen, Ibuprofen, and Triclosan Adsorption. *RSC Adv* **2017**, *7*, doi:10.1039/c7ra08812d.
224. Liu, B.; Sun, Y.; Zheng, T.; He, X.; Wang, P.; Wang, J. Regeneration of Carbon Nanotube Saturated with Tetracycline by Microwave-Ultraviolet System: Performance and Degradation Pathway. *Chemical Engineering Journal* **2020**, *394*, doi:10.1016/j.cej.2020.124752.
225. Wang, J.; Peng, X.; Luan, Z.; Zhao, C. Regeneration of Carbon Nanotubes Exhausted with Dye Reactive Red 3BS Using Microwave Irradiation. *J Hazard Mater* **2010**, *178*, doi:10.1016/j.jhazmat.2010.01.112.
226. Mengelizadeh, N.; Pourzamani, H. Adsorption of Reactive Black 5 Dye from Aqueous Solutions by Carbon Nanotubes and Its Electrochemical Regeneration Process. *Health Scope* **2020**, *9*, doi:10.5812/jhealthscope.102443.
227. Cui, C.; Zheng, Q.; Han, Y.; Xin, Y. Rapid Microwave-Assisted Regeneration of Magnetic Carbon Nanotubes Loaded with p-Nitrophenol. *Appl Surf Sci* **2015**, *346*, doi:10.1016/j.apsusc.2015.03.212.
